# Vaginal microbial dynamics and pathogen colonization in a humanized microbiota mouse model

**DOI:** 10.1038/s41522-023-00454-9

**Published:** 2023-11-20

**Authors:** Marlyd E. Mejia, Vicki Mercado-Evans, Jacob J. Zulk, Samantha Ottinger, Korinna Ruiz, Mallory B. Ballard, Stephanie W. Fowler, Robert A. Britton, Kathryn A. Patras

**Affiliations:** 1 Department of Molecular Virology and Microbiology, Baylor College of Medicine, Houston, TX USA; 2 Medical Scientist Training Program, Baylor College of Medicine, Houston, TX USA; 3 Center for Comparative Medicine, Baylor College of Medicine, Houston, TX USA; 4 Alkek Center for Metagenomics and Microbiome Research, Baylor College of Medicine, Houston, TX USA

**Keywords:** Microbiome, Pathogens

## Abstract

Vaginal microbial composition is associated with differential risk of urogenital infection. Although *Lactobacillus* spp. are thought to confer protection against infection, the lack of in vivo models resembling the human vaginal microbiota remains a prominent barrier to mechanistic discovery. Using 16S rRNA amplicon sequencing of C57BL/6J female mice, we found that vaginal microbial composition varies within and between colonies across three vivaria. Noting vaginal microbial plasticity in conventional mice, we assessed the vaginal microbiome of humanized microbiota mice (^HMb^mice). Like the community structure in conventional mice, ^HMb^mice vaginal microbiota clustered into community state types but, uniquely, ^HMb^mice communities were frequently dominated by *Lactobacillus* or *Enterobacteriaceae*. Compared to conventional mice, ^HMb^mice were less susceptible to uterine ascension by urogenital pathobionts group B *Streptococcus* (GBS) and *Prevotella bivia*. Although *Escherichia* and *Lactobacillus* both correlated with the absence of uterine GBS, vaginal pre-inoculation with exogenous ^HMb^mouse-derived *E. coli*, but not *Ligilactobacillus murinus*, reduced vaginal GBS burden. Overall, ^HMb^mice serve as a useful model to elucidate the role of endogenous microbes in conferring protection against urogenital pathogens.

## Introduction

The vaginal microbiota is inextricably tied to women’s urogenital health. Vaginal microbial perturbations are implicated in risk of adverse outcomes including preterm birth, pelvic inflammatory disease, urinary tract infections, and sexually transmitted infections^1–9^. Across geographic, ethnic, and social demographics, the vaginal microbiota in reproductive-age women is largely comprised of *Lactobacillus* spp. Benefits from *Lactobacillus* spp. include lactic acid-mediated vaginal acidification, hydrogen peroxide and bacteriocin production, competitive adherence at the vaginal epithelium, and immunomodulatory activity^10–16^. While *Lactobacillus* dominance is canonically considered a hallmark of vaginal health, approximately 1 in 5 women have a more diverse vaginal microbiota with a greater proportion of facultative or strictly anaerobic taxa including *Gardnerella vaginalis*, *Candidatus Lachnocurva vaginae* (BVAB1), and *Prevotella*^17,18^. Frequently, women with non-*Lactobacillus* dominant communities are asymptomatic; however, some women experience symptoms such as abnormal discharge and odor and are diagnosed with bacterial vaginosis (BV), while others experiencing inflammation, elevated pH, and overgrowth of aerobic bacteria are diagnosed with aerobic vaginitis (AV)^19,20^. These more diverse communities have gained a reputation as non-optimal due to their association with vaginal symptoms and heightened risk of obstetric and gynecologic complications^18,21–24^.

Although disease associations with human vaginal microbial compositions and insights into specific protective mechanisms have recently expanded, many questions remain regarding microbe-microbe interactions and their interplay with vaginal physiology and host immunity. A prominent barrier to mechanistic discovery is the lack of an in vivo model system resembling the human vaginal microbiota. While mouse models have served a seminal role in delineating host-microbe interactions in reproductive diseases, the murine vaginal microbiota has only recently been defined. Although community structures (low richness and evenness) are similar to humans, the murine phylogenetic composition is quite distinct; *Staphylococcus succinus* and *Enterococcus* spp. are the most frequent taxa in C57BL/6J mice^25,26^, *Enterobacteriaceae* and *Proteus* spp. are dominant in CD-1 mice^27^, and *Streptococcus* spp. and *Proteus* spp. are observed in FVB mice^28^. Conventional mice are poorly colonized by human vaginal *Lactobacillus* spp. and require multiple, high-inoculum doses to observe in vivo effects^29–31^. Several studies have evaluated the ability of human vaginal communities to colonize mice with low or no endogenous microbiota, but stable colonization by these communities was not achieved^32,33^. Not only is there a need to understand vaginal microbial dynamics in conventional mice for disease modeling, but there is also dire need for an animal model that better recapitulates the human vaginal microbiota to provide translational relevance^34,35^.

Here, we evaluated the impact of environment on the vaginal microbiome by comparing conventional C57BL/6J mice raised at three distinct vivaria. To circumvent challenges with transient colonization of human vaginal microbes, we assessed whether a mouse model stably colonized with human fecal microbes, humanized microbiota mice (^HMb^mice)^36^, would exhibit a more human-like vaginal composition. We profiled vaginal communities over multiple cohorts, assessed the impact of estrous on ^HMb^mice vaginal microbiome composition, and determined susceptibility to vaginal colonization by three urogenital pathobionts. We found that the murine vaginal microbiota is malleable in composition and that the distinct ^HMb^mouse vaginal microbiota confers protection against group B *Streptococcus* and *Prevotella bivia* compared to mice with conventional vaginal microbiota.

## Results

### The vaginal microbiota differs between vivaria and demonstrates high intra-colony variability

Human and mouse studies have shown that, despite host genetic selection for certain microbiome features^37^, environmental factors play a dominant role in gut microbial composition^38–45^. It remains unknown whether the vaginal microbiota is likewise affected. To test this, non-pregnant conventional C57BL/6J mouse vaginal swabs were collected from three different sources [Jackson Lab and in-house colonies at Baylor College of Medicine (BCM) and the University of California San Diego (UCSD)] and subjected to 16S v4 rRNA amplicon sequencing. BCM and UCSD samples were sequenced at their respective institutions, with Jackson samples sequenced at both sites. Sequencing depth and reference-mapping was comparable across sites (Supplementary Fig. 1a, b). Unrarefied, BCM-sequenced Jackson communities were more rich than other groups, contributing to increased alpha diversity (Supplementary Fig. 1c, d) and Bray-Curtis dissimilarity compared to UCSD-sequenced Jackson mice (Supplementary Fig. 1e). However, neither the centroids nor dispersion were significantly different between Jackson mice sequenced at either site as determined by PERMANOVA and PERMDISP analyses performed on weighted normalized UniFrac distances (Supplementary Fig. 1f). Upon repeated rarefaction^46,47^, BCM-sequenced and UCSD-sequenced Jackson mice remained clustered by normalized weighted UniFrac distances indicating similar phylogeny and feature abundance (Supplementary Fig. 1g). Jackson mice were therefore grouped in the following analyses. To retain samples and capture the full diversity of the vaginal communities, sequences were filtered of contaminants without use of rarefaction (Supplementary Fig. 2).

**Fig. 1:**
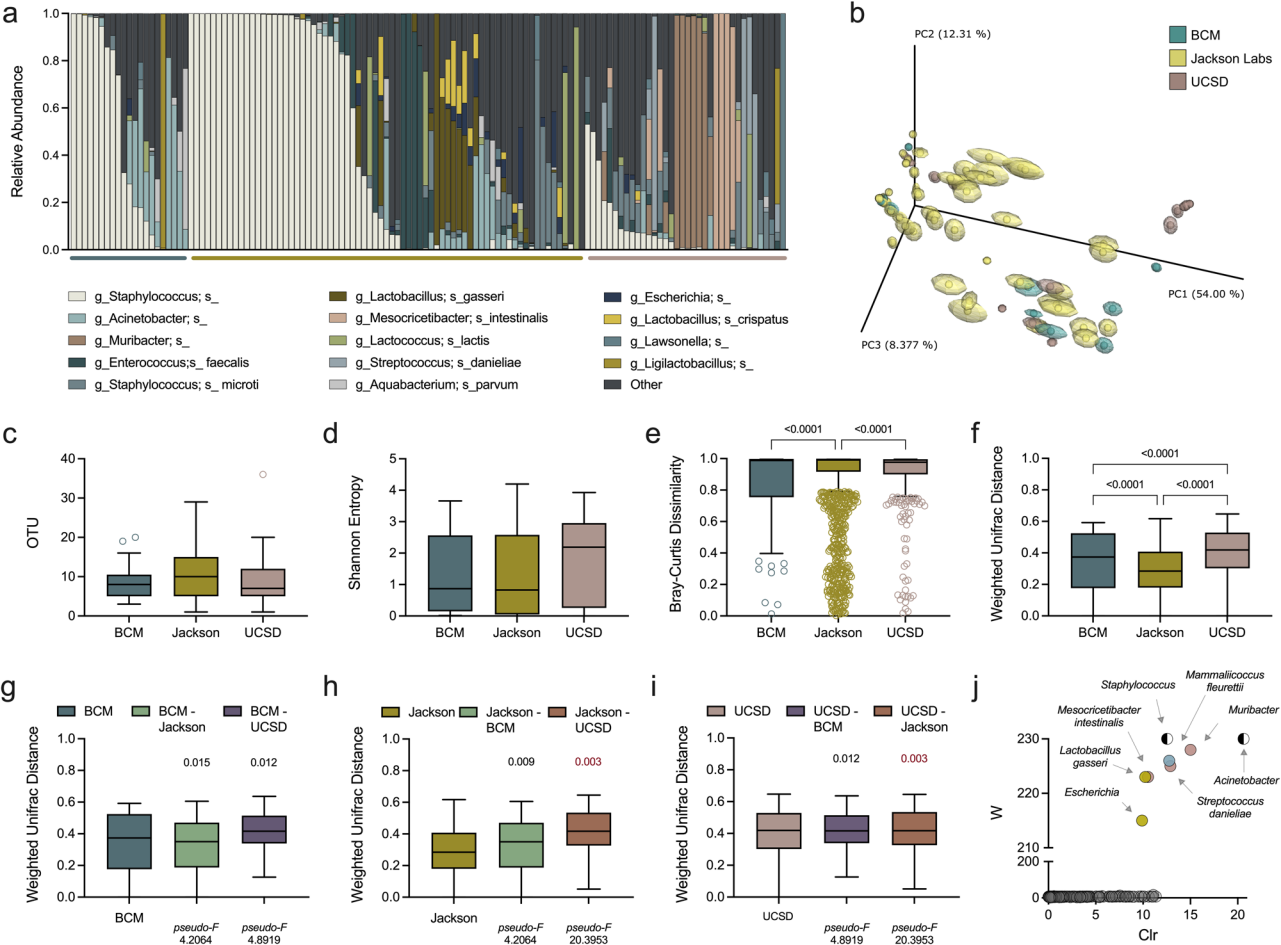
The microbial composition of the murine vaginal tract varies within colonies and between vivaria. Vaginal swabs were collected from mouse colonies raised at BCM (*n* = 21), Jackson Lab (*n* = 71), and UCSD (*n* = 36, 12 mice repeatedly swabbed). **a** Vaginal microbial compositions of mice at BCM (blue bar), Jackson Lab (yellow bar), and UCSD (pink bar). **b** PCoA of vaginal communities clustered by weighted normalized UniFrac distances between mice at different vivaria. Centroids were determined by jackknifed rarefaction (100 reads). **c** Observed OTUs and (**d**) Shannon entropy of vaginal swab samples. **e** Bray-Curtis and (**f**) weighted normalized UniFrac distances between murine vaginal communities of mice within colonies (intra-site) at BCM, Jackson Lab, or UCSD. Colony instability and inter-site variation (weighted normalized UniFrac distances) in comparison to (**g**) BCM, **(h)** Jackson Labs, and (**i**) UCSD. **j** ANCOM results identifying unique taxa according to colony origin colored by BCM (blue), Jackson Lab (yellow), and UCSD (pink), or BCM and Jackson Lab (black and white). Each column (**a**) or symbol (**b**) represents a unique vaginal swab community. Tukey’s boxplots are displayed (**c**−**i**). Data were analyzed by Kruskal-Wallis with a Dunn’s multiple comparison test (**c**−**f**) or PERMANOVA followed by PERMDISP (**g**−i). PERMANOVA *P* values (*P* < 0.1) are colored in red if PERMDISP was also statistically significant (*P* < 0.05). All statistically significant *P* values are reported.

BCM, Jackson, and UCSD mice had similar community structure: 67%, 59% and 44% of mice respectively had vaginal communities dominated (>50% relative abundance) by a single taxon (Fig. 1a). Colonies also contained shared and unique taxa. *Staphylococcus-*dominant communities were present in all three colonies but at differing proportions: 9/21 (43%), 30/71 (42%) and 2/36 (5.6%) of BCM, Jackson, and UCSD samples, respectively (Fig. 1a). Weighted normalized UniFrac distances calculated after repeated rarefaction demonstrated overlap in community composition across colonies, but with several unique clusters of Jackson and UCSD mice (Fig. 1b). Mice at all three sites had similar vaginal OTUs and Shannon diversity (Fig. 1c, d). Intra-colony Bray-Curtis dissimilarity scores indicated that the Jackson colony had the lowest commonality among mice, although all three groups had diverse compositions within their respective colonies (Fig. 1e). Phylogenetically, however, UCSD samples showed more compositional diversity while Jackson samples clustered more closely to one another (Fig. 1f, Supplementary Fig. 1g).

Inter-colony comparisons demonstrated dissimilar compositions between colonies through multiple metrics including Bray-Curtis dissimilarity (Supplementary Fig. 1e, Supplementary Fig. 3) and weighted normalized UniFrac distances (Fig. 1g−i, Supplementary Fig. 1f) with Jackson and UCSD samples demonstrating the greatest inter-colony dissimilarity [weighted normalized UniFrac _*pseudo-F*_ = 20.3953, *P* = 0.003). Taxonomic drivers of colony distinctions were identified by ANCOM and included *Muribacter, Streptococcus danieliae* and *Mesocricetibcacter intestinalis* in UCSD samples, *Mammaliicoccus fleurettii* in BCM samples, and *Lactobacillus gasseri* and *Escherichia* in Jackson samples (Fig. 1j). *Staphylococcus* and *Acinetobacter* distinguished BCM and Jackson samples from UCSD. Together, these data support that vaginal microbial composition in the C57BL/6J genetic background retains a core community structure, but demonstrates unique taxonomic features that are subject to environmental influence.

### ^HMb^mice have distinct vaginal communities compared to conventional mice and are enriched in *Lactobacillus*-dominant communities in a cohort-specific manner

**Fig. 2:**
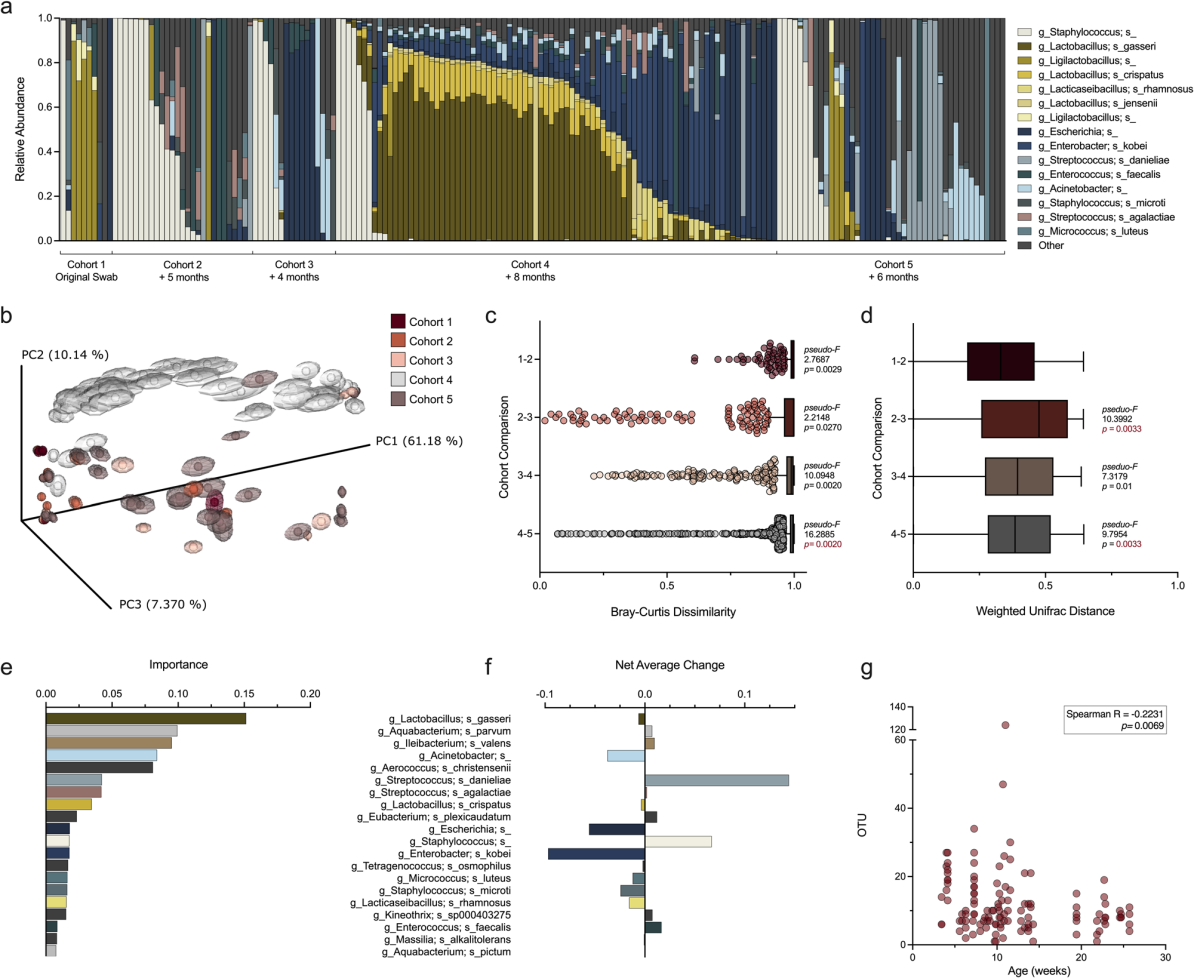
^HMb^mouse vaginal microbiota contains distinct taxa compared to conventional mice and is dynamic within the colony and over time. Vaginal swabs were collected from separate cohorts of ^HMb^mice over the course of two years. **a** Vaginal microbial compositions of distinct cohorts with the duration since previous sampling noted below. Samples from Cohort 1 – Cohort 3 (*n* = 10−27) represent baseline vaginal swabs from unique mice. Cohort 4 and 5 (*n* = 12−23) were swabbed repeatedly at baseline over the course of a week; all samples are displayed. **b** PCoA of vaginal communities clustered by weighted normalized UniFrac distances between mice in different cohorts. Centroids were determined by jackknifed rarefaction (100 reads). Dissimilarity between consecutive cohorts using (**c**) Bray-Curtis and (**d**) weighted normalized UniFrac distances. **e** Top 20 important features over the course of repeated sampling of Cohort 4 and 5 and (**f**) the net average change in feature relative abundance. **g** Changes in vaginal community OTU with increased age. Each column (**a**) or symbol (**b**, **g**) represents a unique vaginal swab community. Tukey’s boxplots are displayed (**c**−**d**). Values for each feature are displayed (**e**−**f**) and colored as in (**a**). Data were statistically analyzed by PERMANOVA followed by PERMDISP (**c**−**d**), Random Forest Regression on 50 estimators (**e**), and Spearman Correlation (**g**). PERMANOVA *P* values (*P* < 0.1) are colored in red if PERMDISP was also statistically significant (*P* < 0.05). All statistically significant *P* values are reported.

To determine whether stable colonization of human-derived microbes in mice would alter the vaginal microbiota, we defined the vaginal microbiome of ^HMb^mice. ^HMb^mice, founded from germ-free WT C57BL/6J mice colonized with human fecal microbiota, display more human-like gastrointestinal communities compared to conventionally-raised mice and demonstrate generational stability among offspring^36^. Vaginal swabs collected from multiple generations of ^HMb^mice were subjected to 16S rRNA v4 amplicon sequencing. Half of the first cohort of mice, twelve generations removed from founder mice, demonstrated *Lactobacillus* dominance (Fig. 2a). Subsequent cohorts had at least one mouse with *Lactobacillus* colonization (0.0025−99% relative abundance), though the incidence of *Lactobacillus*-dominant mice was cohort-specific (Fig. 2a). Importantly, vaginal Lactobacilli in ^HMb^mice represent multiple species according to 16S v4 sequences. The most frequent *Lactobacillus* sequences identified by Greengenes2^48^ mapped to *L. gasseri, L. crispatus*, or *L. jensenii* (Fig. 2a). *Ligilactobacillus* sequences mapped to *L. animalis and L. murinus* (Supplementary Table 1), which have been isolated from the murine gastrointestinal and vaginal tracts, the latter of which has also been detected in vaginal tract of women who work in rural settings^49–52^. Paired vaginal and fecal microbiota of ^HMb^mice (Cohort 3) shared few taxa (e.g., *L. reuteri, L. gasseri*, and *Ligilactobacillus*), but remained compositionally distinct, clustering by sample type on a rarefied PCoA generated from weighted normalized UniFrac distances (Supplementary Fig. 4a, b). Vaginal samples had decreased richness and lower Shannon entropy compared to fecal pellets in unpaired (Supplementary Fig. 4c, d) and paired analyses (Supplementary Fig. 4e). There was no correlation in Shannon entropy between paired fecal and vaginal communities (Supplementary Fig. 4f). Additionally, community variability among site-specific samples was higher in vaginal compared to fecal communities (Supplementary Fig. 4g), and vaginal communities were more phylogenetically diverse than fecal compositions (Supplementary Fig. 4h). Together, these data demonstrate site-specific colonization of human fecal microbes in a murine model.

Vaginal microbial alpha diversity was similar in ^HMb^mice and conventional mice (Supplementary Fig. 5a, b), and Bray-Curtis dissimilarity comparisons showed high ^HMb^mouse intra- and inter-colony dissimilarities (Supplementary Fig. 5c). Phylogenetically, ^HMb^mice were most distinct from UCSD mice and Jackson mice (Supplementary Fig. 5d). Overlaid PCoA visualization of ^HMb^mice and conventional mice showed overlap of ^HMb^mice and Jackson samples that was partially driven by *Lactobacillus* spp. and supports that differences in centroids, not dispersion, primarily drove colony variation (Supplementary Fig. 5d, e). In contrast, differences between ^HMb^mice and BCM mice were influenced by dispersion (Supplementary Fig. 5c). Low vaginal pH is a hallmark of a *Lactobacillus*-rich environment in humans^53^; vaginal lavage pH was slightly, but significantly lower in ^HMb^mice (median 6.33) compared to conventional mice (median 6.53)(Supplementary Fig. 5f). To determine whether ^HMb^mice were more permissive to human vaginal species, *L. crispatus Strep*^*R*^ was intravaginally administered to *β*-estradiol synchronized mice. Recovered levels of *L. crispatus* were not significantly different between conventional BCM and ^HMb^mice across five days post-inoculation (Supplementary Fig. 5g).

Within the ^HMb^mice colony, ANCOM analysis identified *Acinetobacter, L. gasseri, E. faecalis, L. crispatus, L. jensenii, E. kobei, L. rhamnosus, Escherichia, A. christensenii, B. xylanisolvens, L, fermentum, S. anginosis, S. danieliae*, and *B. longum* as the greatest drivers of cohort variation. Indeed, *L. gasseri* and *E. kobei* were most prevalent in Cohort 4 and drove clustering by weighted normalized UniFrac distances (Fig. 2b). Interestingly, the extent of dissimilarity between cohorts did not correspond with time elapsed between sampling periods. For example, phylogenetic diversity between vaginal samples from Cohort 2 and 3 taken four months apart (*pseudo-F* = 10.3992) was greater than that between Cohort 3 and 4 taken eight months apart (*pseudo-F* = 7.3179)(Fig. 2c, d). Significant differences in dispersion were detected between Cohort 2 and 3, and Cohort 4 and 5, but not Cohort 3 and 4 (denoted by the red coloring of the PERMANOVA *P-*value)(Fig. 2d).

We also examined vaginal composition dynamics over time in a subset of mice (Cohorts 4 and 5) recently transferred between rooms. Random Forest Regression analysis of samples collected daily or every three days for a week revealed that *L. gasseri* was the most important taxa (feature) (Fig. 2e). Despite *L. gasseri* having the greatest average increase in frequency, the net average change in features ranged from a slight decrease in *L. gasseri*, to larger relative changes in *E. kobei* and *S. danieliae* (Fig. 2f). To evaluate changes to the vaginal community with age, we compared alpha diversity across baseline samples and found that observed OTUs decreased as age of mice increased (Fig. 2g).

### Reproductive parameters vary across ^HMb^mice, conventional, and germ-free colonies

Vaginal microbial composition is implicated in birth outcomes in humans^54,55^ and fecundity in mice; reproductive success improves when germ-free mice become colonized with bacteria^56^. To resolve whether ^HMb^mice have altered reproductive capacity, ^HMb^mice colony reproductive data were compared against conventional C57BL/6J mice at Jackson Lab^57^ and genetically-matched conventional and germ-free mice at BCM. Dams in the ^HMb^mice colony averaged 14.4 weeks when their first litter was successfully weaned, which was significantly older than Jackson and BCM mice, but younger than germ-free BCM mice (*P* < 0.0001 for all comparisons)(Table 1). ^HMb^dams had an average of 3.5 total litters, falling below germ-free mice, conventional mice, and Jackson mice; however, both starting and terminating breeding ages are in part determined by colony management staff. The average projected number of per-dam litters over six months, on the other hand, indicated that ^HMb^mice (5.1 litters) resembled conventional BCM mice (4.9 litters) and could theoretically produce more litters than germ-free and Jackson mice. The gestational interval in ^HMb^mice averaged at 6.8 weeks, aligning more to the timeline of germ-free mice. The average litter size of ^HMb^mice (5.8 pups) was similar to Jackson and conventional BCM mice (5.9 and 5.6 respectively), and significantly higher than germ-free BCM mice (5.0 pups). Together, ^HMb^mice breeding frequency falls within the range observed for conventional and germ-free mice while litter size most resembles Jackson mice, implying that a humanized microbiota minimally affects reproductive performance.

**Table 1:** Reproductive parameters^a^ for C57BL/6J mice housed in different facilities and colonized by different microbial communities.

	^HMb^mice (BCM)	Conventional mice (Jackson Labs)^b^	*p*- value	Conventional mice (BCM)	*p-* value	Germ-free mice (BCM)	*p-* value
Age of dam at first productive litter (weeks)^c^	14.4 [7.4−35.6]	9.6	<0.0001	12.6	<0.0001	20.0 [7−39]	<0.0001
Total # of litters per dam	3.5 [1−8]	5.4	<0.0001	4.8	<0.0001	3.8	0.0263
Litters (total #/6 months)	5.1 [3−7]	4.0	<0.0001	4.9 [3−6]	0.1459	4.7 [1−6]	0.0054
Gestational interval (weeks)	6.8 [2.9−19.3]	Not specified	*-----*	4.6 [3−14.3]	<0.0001	6.8 [3−12.6]	0.7158
Litter Size	5.8 [1−18]	5.9	0.1520	5.6 [1−11]	0.1183	5.0 [2−9]	<0.0001

^a^Mean values and ranges (where available) are reported.

^b^Data for Jackson Labs were taken from The Jackson Laboratory Handbook on Genetically Standardized Mice, 6th edition^57^.

^c^First litters are typically lost despite being a productive mating. First successfully weaned litters are reported for BCM colonies.

### The vaginal microbiota in ^HMb^mice is minimally influenced by estrous stage

**Fig. 3:**
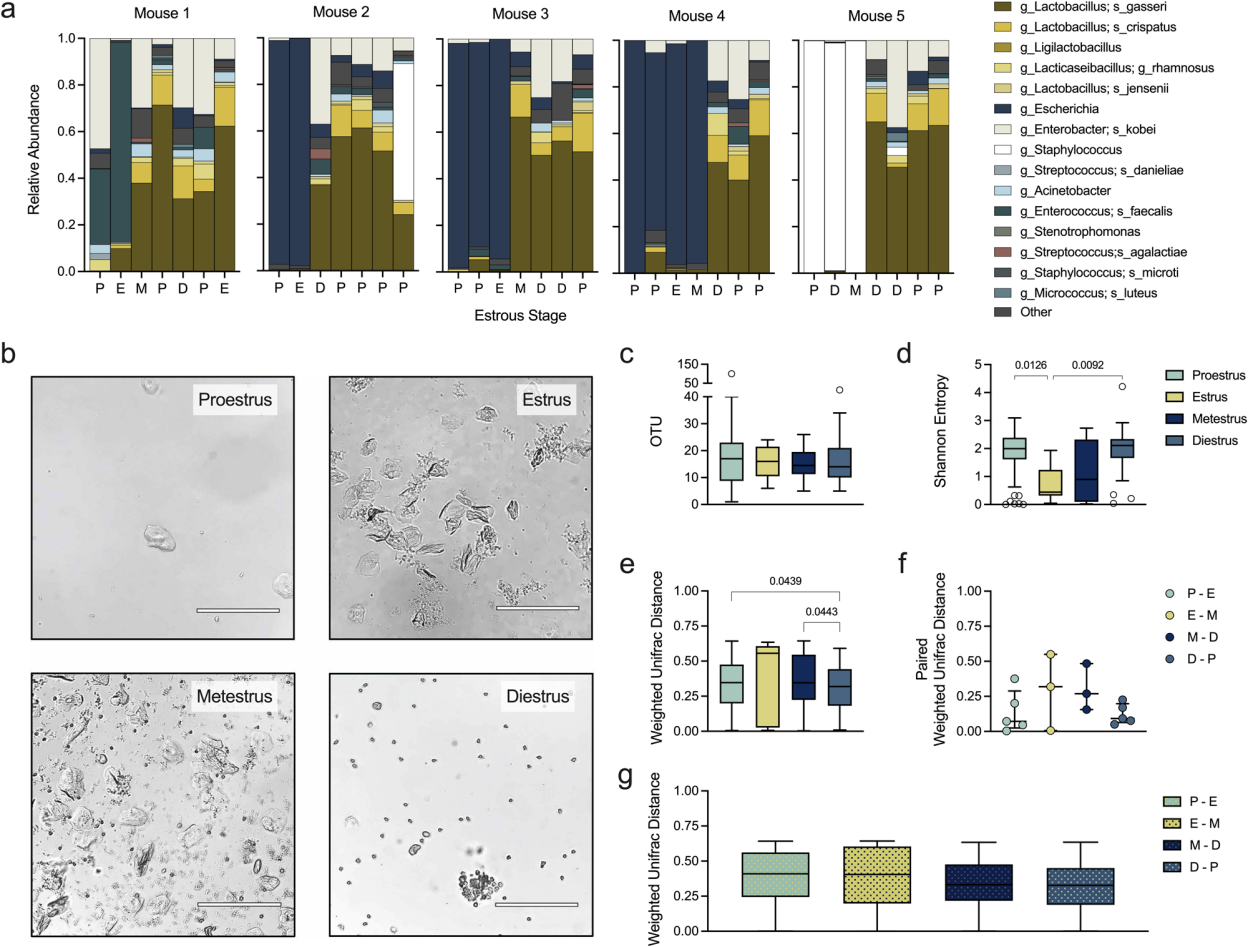
Vaginal microbiota dynamics across estrous stages in ^HMb^mice. Vaginal swabs were collected at baseline and assigned the host’s estrous stage at time of collection. Estrous stage assignment is denoted as P = proestrus, E = estrus, M = metestrus, D = diestrus. **a** Vaginal microbial compositions of five individual ^HMb^mice from Fig. 2 swabbed daily over the course of a week. Only Mouse 1 and Mouse 2 were co-housed. **b** Representative microscopic images of vaginal wet smears collected at each stage of the estrous cycle using a 10X objective lens on a brightfield microscope. Scale bar shows 150 μm. **c** Observed OTUs and (**d**) Shannon diversity of vaginal swab samples (*n* = 35). **e** Weighted normalized UniFrac distances of microbial compositions among samples categorized in the same estrous stage. **f** Weighted normalized UniFrac distances of microbial compositions between paired, consecutive samples collected from individual mice. **g** Weighted normalized UniFrac distances of microbial compositions between unpaired samples from sequential estrous stages. Tukey’s boxplots (**c**−**e**, **g**) or individual comparisons marked as symbols (**f**) are displayed with median and interquartile ranges. Data were statistically analyzed by Kruskal-Wallis with Dunn’s multiple comparisons test (**c**−**f**) or PERMANOVA followed by PERMDISP (**g**). PERMANOVA *P* values (*P* < 0.1) are colored in red if PERMDISP was also statistically significant (*P* < 0.05). All statistically significant *P* values are reported.

The human vaginal microbiota moderately fluctuates over the course of the menstrual cycle as alpha diversity increases and *Lactobacillus* relative abundance decreases during menses^58–63^. To determine if vaginal composition in ^HMb^mice is influenced by estrous stage, five ^HMb^mice were swabbed daily for one week. Over the one-week time course, each mouse displayed at least two different dominant (>50% relative abundance) taxa (Fig. 3a). Estrous stages were then assigned by visualizing wet smears of vaginal samples as described previously^26^(Fig. 3b) and matched to sequencing data for the five mice in Fig. 3a and an additional 30 mice that were sampled periodically. While richness did not differ between stages (Fig. 3c), Shannon entropy was lower in estrus than in both proestrus and diestrus (Fig. 3d). Bray-Curtis dissimilarity approached 1 (highly dissimilar) for all stages, dipping slightly, but significantly, between mice during the diestrus phase compared to proestrus and metestrus (Supplementary Fig. 6a). Parallel results were obtained when comparing weighted normalized UniFrac distances (Fig. 3e). When consecutive transitions from one stage to the next were paired by individual mice, communities transitioning to and from diestrus appeared more similar than in other transition stages, but did not reach statistical significance (Fig. 3f, Supplementary Fig. 6b). Though variability in the vaginal microbiota occurred within each stage, vaginal compositions did not fluctuate drastically between consecutive stages (Fig. 3g, Supplementary Fig. 6c). Together, these results suggest gradual community convergence and increased community stability within mice during diestrus.

### The vaginal microbiota in ^HMb^mice clusters into unique community state types

**Fig. 4:**
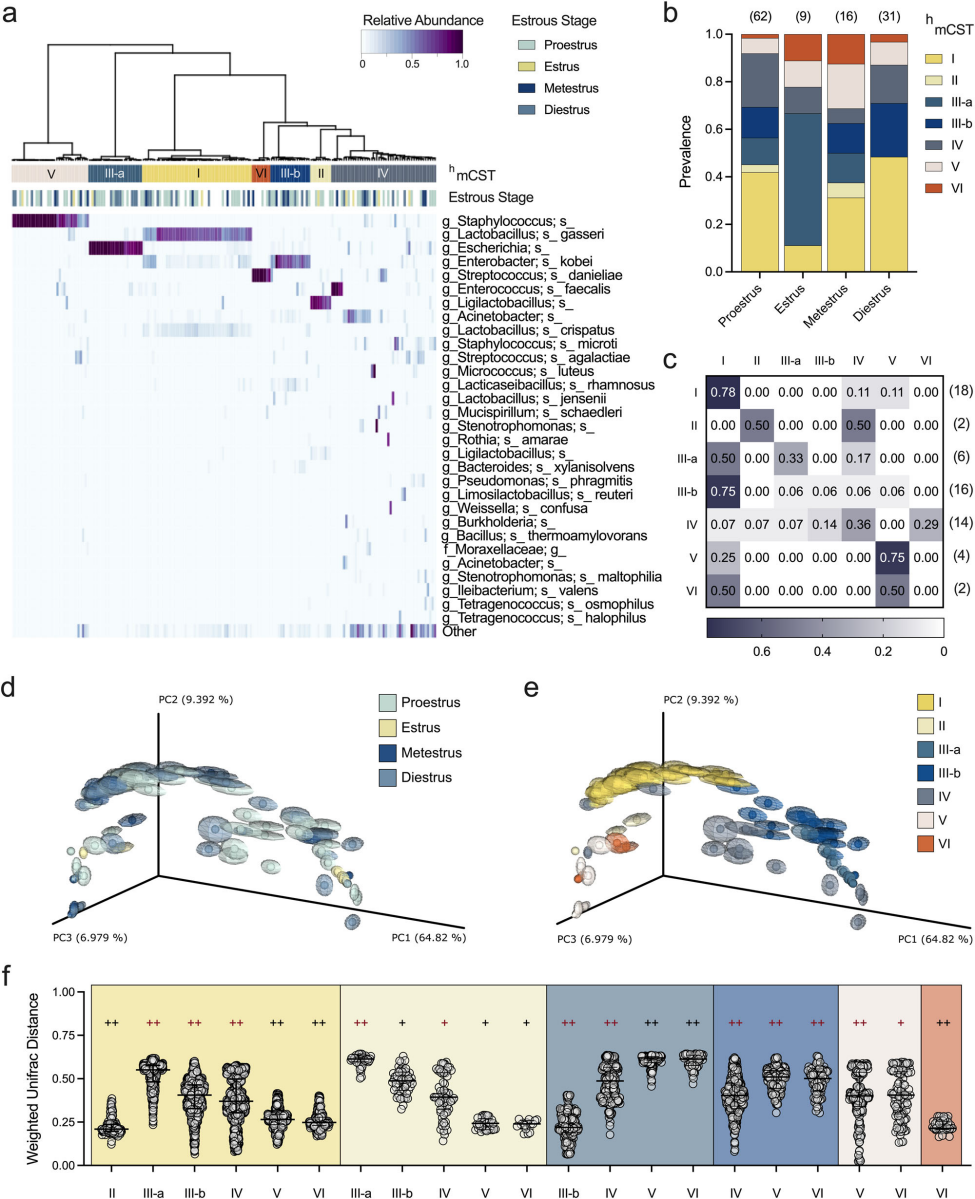
^HMb^mice exhibit distinct community state types that vary in frequency by estrous stage. Vaginal swabs were collected and categorized by both estrous stage and composition. **a** Community state type categorization for ^HMb^mice (*n* = 183 samples) hierarchically clustered into humanized murine CST (upper bar) and its associated estrous stage (lower bar). Estrous staged (*n* = 35) ^HMb^mice from Figs. 2 and 3 and unstaged (*n* = 64) ^HMb^mice from Fig. 2 are depicted. **b** Prevalence of ^h^mCSTs in each estrous stage with total samples per stage noted above. **c** ^h^mCST transition rates from starting community (left) to community three days later (top) with total numbers per starting ^h^mCST noted on the right. PCoA of vaginal communities clustered by weighted normalized UniFrac distances between mice assigned different (**d**) estrous stage and (**e**) ^h^mCST. Centroids were determined by jackknifed rarefaction (100 reads). **f** Weighted normalized UniFrac distances of microbial compositions between ^h^mCSTs. ^h^mCSTs labeled below are compared to the ^h^mCST of the corresponding background color. Each column (**a**) or symbol (**d**−**e**) represents a unique vaginal swab community. Individual comparisons marked as symbols (**f**) are displayed with median and interquartile ranges. Data were analyzed by Chi Square analysis of fractions (**b**) or PERMANOVA followed by PERMDISP (**f**). PERMANOVA *P* values (*P* < 0.1) are colored in red if PERMDISP was also statistically significant (*P* < 0.05). Statistically significant comparisons are noted in the main text (**b**); ^+^*P* < 0.03; ^++^*P* < 0.003.

Vaginal samples were hierarchically clustered into murine community state types (CST) using Ward’s linkage of Euclidean distances as previously described^25,26^. Because the dominant taxa differed from conventional mice^25,26^, we designated ^HMb^mice profiles as “humanized murine CST” (^h^mCST). Two communities resembled mCSTs of conventional mice; ^h^mCST IV (heterogenous taxa with an even composition) and ^h^mCST V (*S. succinus*-dominant). ^HMb^mice had four unique CSTs: ^h^mCST I and II were *Lactobacillus*-dominant and *Ligilactobacillus*-dominant respectively, ^h^mCST III grouped into two *Enterobacteriaceae*-dominant subsets, and ^h^mCST VI was *Streptococcus*-dominant (Fig. 4a). Proportions of ^h^mCSTs varied significantly between estrous stages (Fig. 4b). Estrus (estrogen-high and progesterone-low) and diestrus (progesterone high) most starkly contrasted: ^h^mCST I was more prevalent in diestrus than estrus (chi square test, *P* = 0.0445) and ^h^mCST III-a was more prevalent in estrus compared to diestrus (*P* < 0.0001), proestrus (*P* = 0.0009), and metestrus (*P* = 0.02). Metestrus displayed increased frequency of ^h^mCST III-a compared to diestrus (*P* = 0.04) and ^h^mCST VI compared to proestrus (*P* = 0.04). In human longitudinal studies, CSTs self-transition (same CST in consecutive time points) between 35–85% of the time^64^, and conventional mice typically maintain their mCST between about 20−80% of the time^25^. Similarly, in ^HMb^mice, self-transitioning frequently occurred in ^h^mCST I and V, whereas ^h^mCST III-b transitioned into ^h^mCST I, and all other communities were less consistent (Fig. 4c). When plotted by weighted normalized UniFrac distances, samples did not separate into discrete clusters based on estrous stage (Figs. 3g, 4d, Supplementary Fig. 6a), but did cluster by ^h^mCST, which is derived from taxonomic classification (Fig. 4e, f, Supplementary Fig. 6d).

### ^HMb^mice exhibit decreased uterine ascension of group B *Streptococcus* compared to conventional mice

**Fig. 5:**
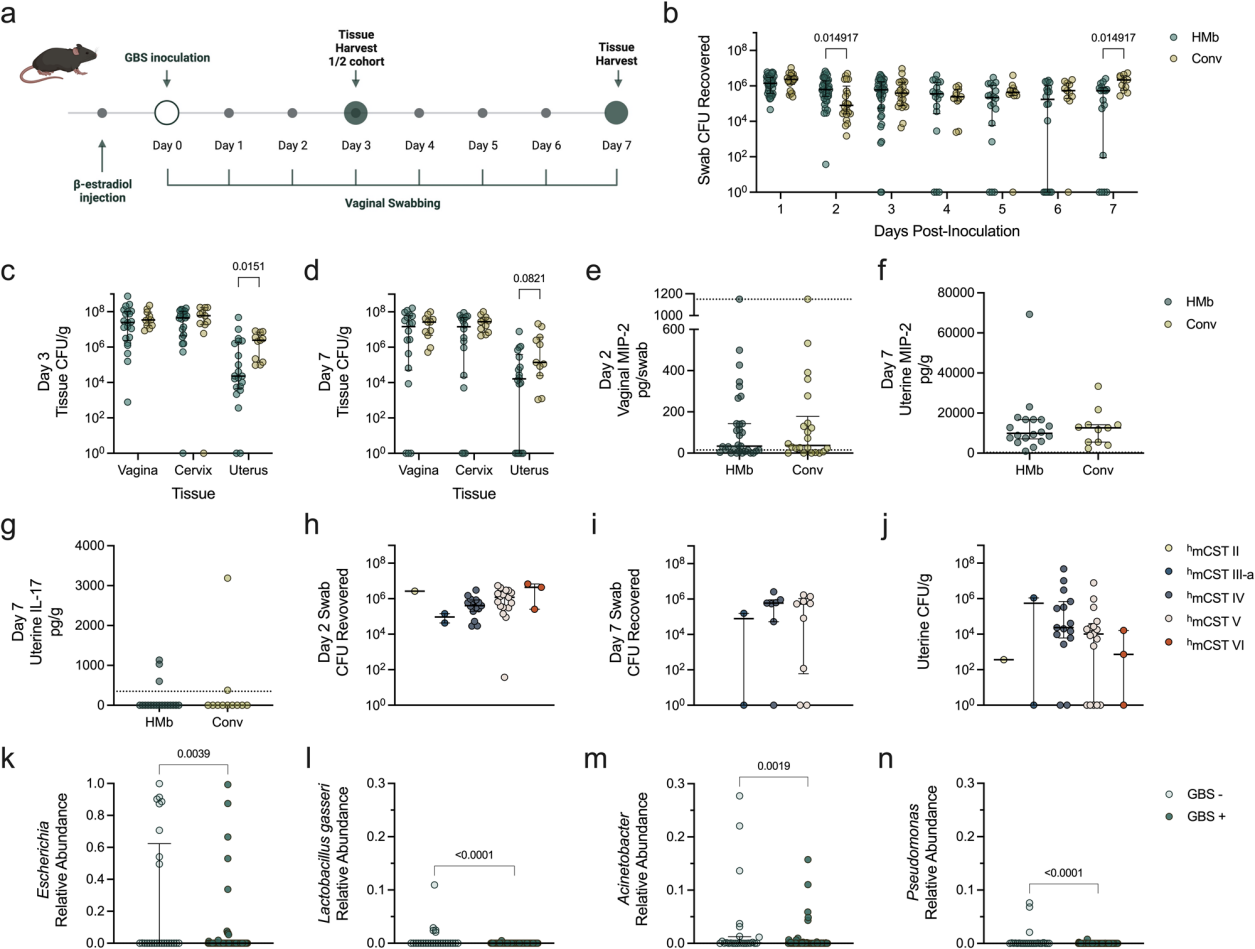
Increased vaginal clearance of GBS and restriction of uterine ascension in ^HMb^mice is not due to ^h^mCST or cytokine levels, but may be attributed to individual taxa. **a** ^HMb^mice (HMb) and conventional (Conv) mice were vaginally inoculated with 10^7^ CFU of GBS (*n* = 10−27). **b** GBS CFU recovered from daily vaginal swabs. Vaginal, cervical, and uterine GBS tissue burdens were collected at (**c**) day 3 and (**d**) day 7 post-inoculation. MIP-2 cytokine levels quantified in (**e**) day 2 vaginal swabs and (**f**) day 7 uterine tissue homogenates, and (**g**) IL-17 quantified in day 7 Uterine homogenates. GBS CFU counts from (**h**) day 2 swabs, (**i**) day 7 swabs, and (**j**) combined day 3 and 7 uterine tissues delineated by ^h^mCST assignment of respective mice on day 0 prior to GBS inoculation. Relative abundances of vaginal (**k**) *Escherichia*, (**l**) *Lactobacillus gasseri*, (**m**) *Acinetobacter*, and (**n**) *Pseudomonas* across all vaginal swabs in mice grouped into detectable uterine GBS (GBS + ) or no detectable uterine GBS (GBS-) at the time of tissue collection. Symbols represent unique swab or tissue samples with median and interquartile ranges. Data were statistically analyzed by Mann−Whitney test (**e**−**g**, **k**−**n**) with corrections for multiple comparisons using the two-stage linear step-up procedure of Benjamini, Krieger and Yekutieli and a false discovery rate (<0.05) for (**b**−**b**), or Kruskal-Wallis with Dunn’s multiple comparison test (**h**−**j**). Statistically significant *P* values are reported. Adjusted *P* values < 0.1 are reported for **b**−**d**. Schematic (**a**) was created with BioRender.com.

To determine whether the distinct ^HMb^mice vaginal microbiota impacted colonization by pathobionts, we used an established group B *Streptococcus* (GBS) colonization model^65^. GBS can asymptomatically colonize the vaginal tract or be associated with AV, a condition characterized by vulvovaginal inflammation and a *Lactobacillus*-depleted vaginal microbiome^19^. Additionally, perinatal exposure to GBS during pregnancy or labor and delivery can cause severe disease including stillbirth or neonatal sepsis^66^. Conventional C57BL/6J mice and ^HMb^mice were vaginally inoculated with GBS (10^7^ CFU) and swabbed daily over seven days (Fig. 5a). At early time points, ^HMb^mice had similar or higher GBS colonization compared to conventional mice. At later time points, ^HMb^mice had significantly lower vaginal GBS burdens than conventional mice with 4/18 ^HMb^mice below the limit of detection for GBS by one-week post-infection (Fig. 5b). To assess GBS ascension, reproductive tract tissues were harvested at day 3 and day 7. Compared to conventional mice, vaginal and cervical GBS burdens were not different between groups, but ^HMb^mice uterine GBS burdens were significantly lower at day 3 and undetectable at day 7 for 6/18 of mice (Fig. 5c, d).

GBS induces vaginal epithelial production of neutrophil-recruiting chemokines including IL-8, or the mouse ortholog MIP-2, during acute exposure^67,68^, and vaginal IL-17 is associated with effective GBS clearance in persistent models of colonization^68^. To determine if these cytokines contributed to the differences between GBS burdens, we measured MIP-2 and IL-17 in day 2 vaginal swab and day 7 uterine tissues by ELISA. In day 2 vaginal swab samples, no differences in vaginal MIP-2 levels were observed between ^HMb^mice and conventional mice (Fig. 5e), and IL-17 levels were below the limit of detection in all samples. In day 7 uterine tissues, no differences were observed in either MIP-2 or IL-17 levels between groups (Fig. 5f, g).

To determine if vaginal microbial composition influenced GBS colonization or ascension, GBS burdens were replotted according to the ^h^mCST assigned at day 0, immediately prior to GBS inoculation. GBS vaginal burdens were not significantly different across ^h^mCST groups at day 2 nor day 7 (Fig. 5h, i). Furthermore, no significant differences in GBS uterine burdens from combined day 3 and 7 samples were detected between ^h^mCSTs (Fig. 5j). To determine whether specific vaginal taxa were associated with GBS uterine ascension, ^HMb^mice were binned into two categories across both time points: those with no detectable GBS uterine CFU (GBS-) or those with detectable GBS uterine CFU (GBS + ). Corresponding vaginal swab 16S sequences from all timepoints were probed for differentially abundant taxa by ANCOM. Mice with no detectable uterine GBS exhibited an enrichment of *Escherichia*, *Lactobacillus*, *Acinetobacter*, *and Pseudomonas* (Fig. 5k−n).

### *Ligilactobacillus murinus* and *E. coli* display discordant phenotypes towards GBS in competition assays in vitro and GBS vaginal colonization in vivo

**Fig. 6:**
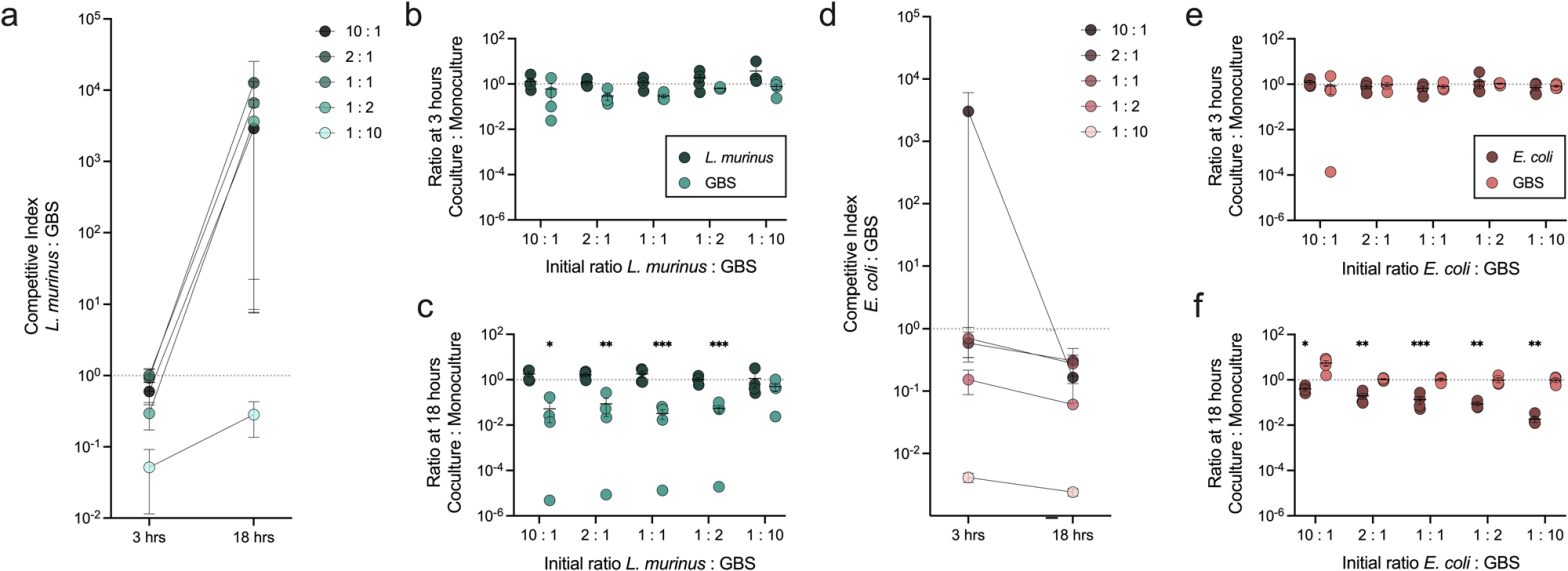
In vitro competition assays demonstrate GBS inhibition by *L. murinus* but not *E. coli*. In vitro competition assays were performed between GBS and (**a**−**c**) *L. murinus* or (**d**−**f**) *E. coli*. **a**, **d** Competitive index (CI) was calculated as the CFU ratio of the treatment microbe over GBS, normalized to the initial ratio of the inoculum. The rate of bacterial growth (viable CFU) in coculture compared to monoculture controls was calculated at timepoints (**b**, **e**) 3 h and (**c**, **f**) 18 h. Symbols represent the mean of four independent experiments shown with the standard error of the mean (s.e.m.) (**a**−**d**) or independent experimental replicates (**b**−**c**, **e**−**f**) with mean and s.e.m. Data were statistically analyzed by one-sample *t-*test with a theoretical mean of 1.0 (**a**, **d**) and two-way ANOVA with Šídák’s multiple comparisons test for coculture deviation from growth in monoculture (**b**−**c**, **e**−**f**); ^*^P < 0.05; ^**^P < 0.005^; ***^P < 0.0005. Raw viable CFU values are reported in Fig. S7.

To gain insight into function of differentially abundant taxa between groups, bacterial isolates were collected from ^HMb^mice. Two isolates representing ^h^mCST II and ^h^mCST III-a communities, identified as *L. murinus* and *E. coli* by full-length 16S sequencing, were each cultured in MRS broth in competition with GBS at two timepoints across five different starting ratios. Minimal differences in competitive index were observed between *L. murinus* and GBS, with GBS displaying a competitive advantage at the 1:2 and 1:10 ratios at 3 h (*P* = 0.01 and 0.0002 respectively) which was retained at 18 h in the 1:10 condition (*P* = 0.017)(Fig. 6a). To determine how coculture impacted growth of each organism, viable CFU of each organism in coculture was compared to CFU recovered from monoculture. *L. murinus* was minimally impacted (Fig. 6b); however, GBS growth at 18 h was impaired in the presence at *L. murinus* at all but the highest GBS starting inoculum (1:10 *L. murinus* to GBS)(Fig. 6c). Conversely, GBS demonstrated a strong competitive advantage in coculture with *E. coli*, which was significant in the 1:2 and 1:10 conditions at 3 h (*P* = 0.001 and <0.0001 respectively) and in all five ratios at the 18 h timepoint (*P* ≤ 0.029)(Fig. 6d). No differences were observed between E. coli growth in co- or monoculture at 3 h (Fig. 6e). At 18 h, *E. coli* growth was significantly impaired in the presence of GBS in all conditions (Fig. 6f). Raw viable CFU values for each organism are provided in Supplementary Fig. 7.

**Fig. 7:**
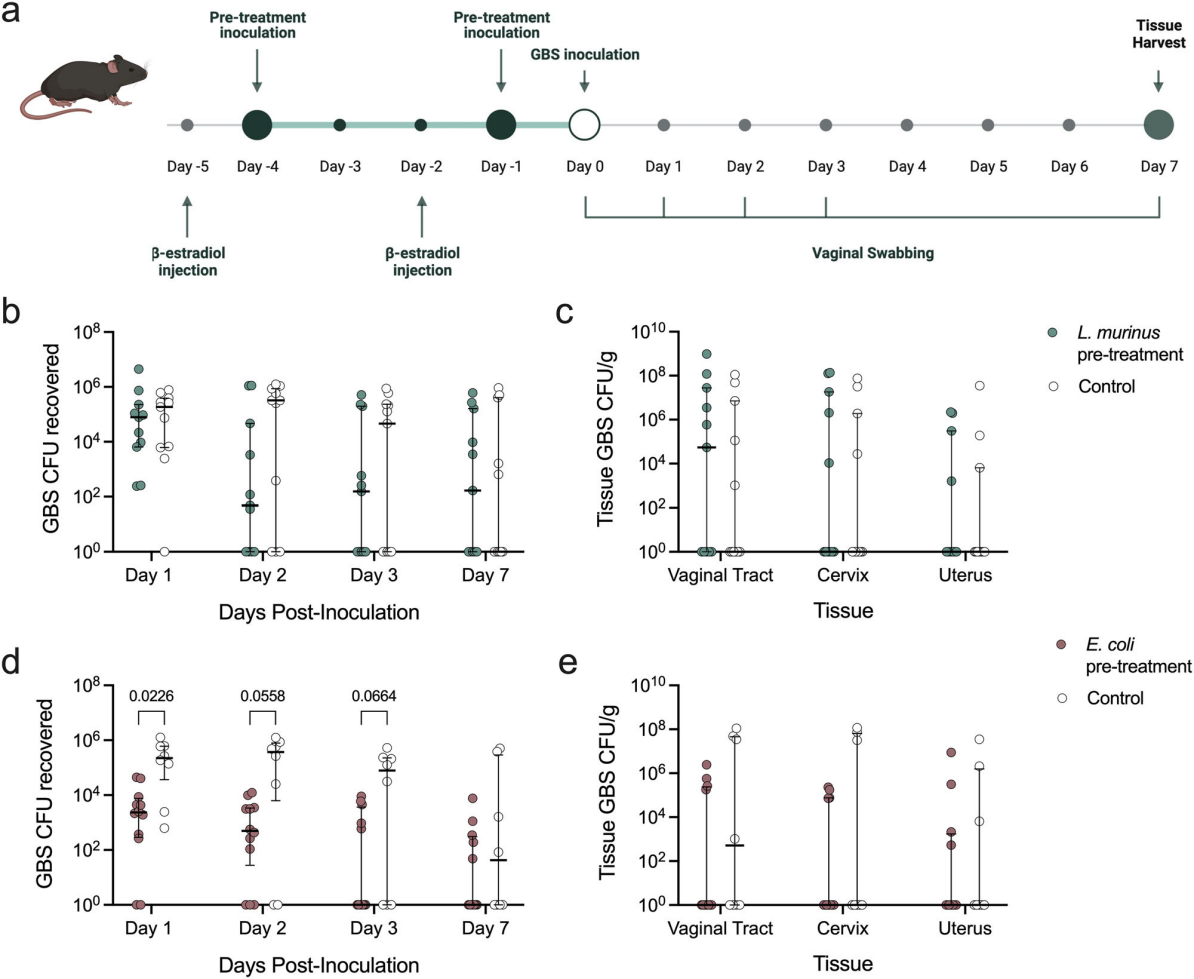
Pretreatment with *E. coli*, not *L. murinus*, reduces GBS vaginal colonization in vivo. **a** ^HMb^mice were vaginally inoculated with 10^7^ CFU of *L. murinus* (*n* = 11/group), 10^7^ CFU of *E. coli* (*n* = 8−12/group), or vehicle control twice prior to challenge with 10^7^ CFU of GBS. Recovered GBS CFU from (**b**) vaginal swabs and (**c**) day 7 reproductive tract tissues of mice pre-inoculated with *L. murinus*. Recovered GBS CFU from (**d**) vaginal swabs and (**e**) day 7 reproductive tract tissues of mice pre-inoculated with *E. coli*. Symbols represent individual mice with median and interquartile ranges. Data were statistically analyzed by Mann−Whitney with corrections for multiple comparisons using the two-stage linear step-up procedure of Benjamini, Krieger and Yekutieli and a false discovery rate (<0.05). Adjusted *P* values < 0.1 are reported. Schematic (**a**) was created with BioRender.com.

To determine whether endogenous strains could be re-introduced to confer protection against GBS, *L. murinus* or *E. coli* were separately vaginally inoculated into ^HMb^mice twice prior to GBS challenge (Fig. 7a). Despite decreased growth of GBS in the presence of *L. murinus* in vitro, GBS vaginal colonization and dissemination into the upper reproductive tract was unaffected in vivo (Fig. 7b, c). Prophylactic inoculation with *E. coli* reduced GBS vaginal burden one day post-inoculation (Fig. 7d) but did not influence swab or tissue burdens by day 7 (Fig. 7e).

### ^HMb^mice exhibit decreased tissue dissemination of *Prevotella bivia*, but not uropathogenic *E. coli*, compared to conventional mice

**Fig. 8:**
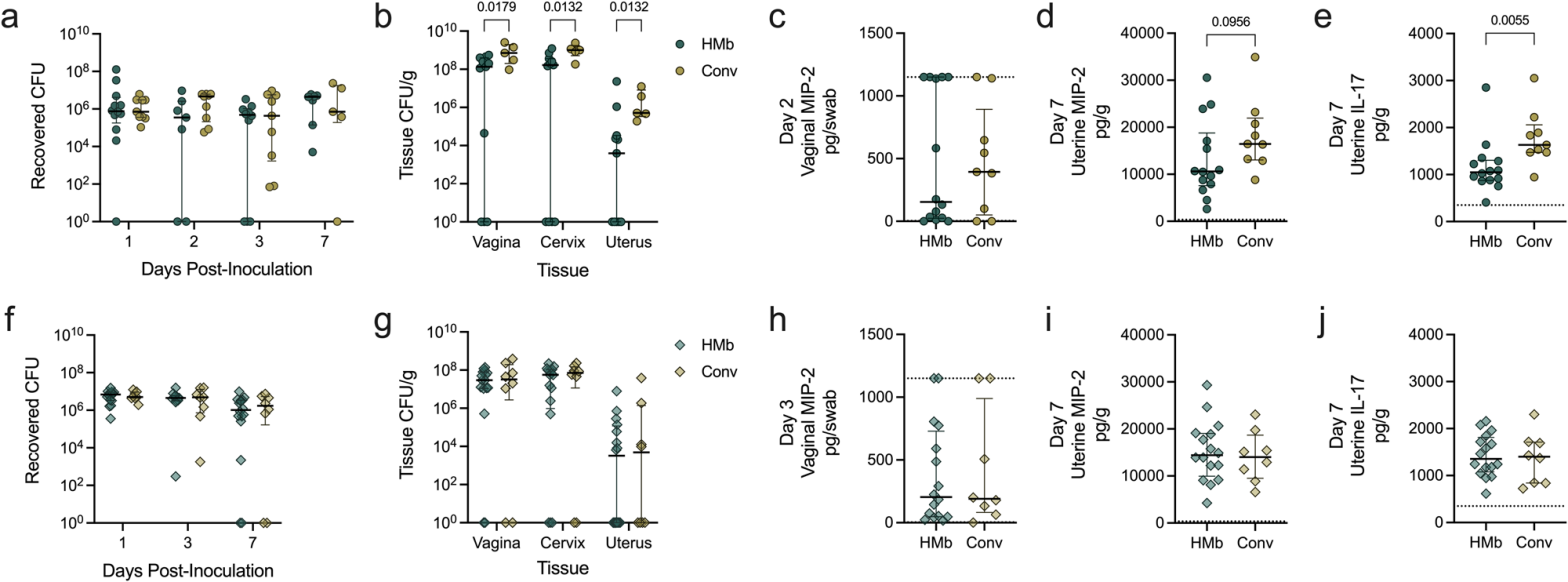
^HMb^mice have reduced cervical and uterine tissue burdens of *P. bivia* but lack protection against colonization or ascension of UPEC. ^HMb^mice (HMb) and conventional (Conv) mice were inoculated with 10^6^ CFU of *P. bivia* (*n* = 9−16) or 10^7^ CFU of UPEC (*n* = 8−16). Recovered *P. bivia* CFU from (**a**) vaginal swabs collected on days 1, 2, 3, and 7 post-inoculation and (**b**) reproductive tract tissues harvested on day 7 (*n* = 5−12). MIP-2 quantified in (**c**) day 2 vaginal swabs and (**d**) day 7 uterine homogenates along with (**e**) IL-17 in day 7 uterine homogenates of *P. bivia-*inoculated mice. Recovered UPEC CFU from (**f**) vaginal swabs collected on days 1, 3, and 7 post-inoculation and (**g**) reproductive tract tissues harvested on day 7. MIP-2 quantified in (**h**) day 2 vaginal swabs and (**i**) day 7 uterine homogenates along with (**j**) IL-17 in day 7 uterine homogenates of UPEC-inoculated mice. Symbols represent individual mice with median and interquartile ranges. Data were statistically analyzed by Mann−Whitney test (c−**e**, **h**−**j**) with corrections for multiple comparisons using the two-stage linear step-up procedure of Benjamini, Krieger and Yekutieli and a false discovery rate (<0.05)(**a**−**b**, **f**−**g**). Statistically significant *P* values are reported. Adjusted *P* values < 0.1 are reported for (**a**−**b**), (**f**−**g**). *P* < 0.1 is shown (**d**) to be transparent of lack of significance.

To determine whether ^HMb^mice were protected against multiple pathogens or selectively resistant to GBS, we challenged ^HMb^mice with two additional pathobionts associated with vaginal dysbiosis: *Prevotella bivia*, which is increased in women diagnosed with BV^69^, and uropathogenic *E. coli* (UPEC), a causative agent for urinary tract infection that can establish vaginal reservoirs^70^ or cause AV^20^. Unlike GBS, there was no difference in *P. bivia* vaginal swab burdens between conventional or ^HMb^mice at any timepoint (Fig. 8a). However, at day 7, *P. bivia* burdens in vaginal, cervical, and uterine tissues were significantly lower in ^HMb^mice compared to conventional mice (Fig. 8b). There were no significant differences in vaginal or uterine MIP-2 (Fig. 8c, d), but IL-17 levels were reduced in ^HMb^mice compared to conventional mice (Fig. 8e). In comparison, no differences in UPEC burdens were observed between ^HMb^mice and conventional mice in vaginal swabs (Fig. 8f) or tissues (Fig. 8g), and cytokine levels were comparable between groups (Fig. 8h−j).

## Discussion

Despite strong clinical correlations between the vaginal microbiota and women’s health outcomes, ascribing function of the microbiota in vaginal health and disease susceptibility is impaired by the lack of an animal model that recapitulates the human vaginal microbiota. *Lactobacillus* spp. dominate ~73% of human vaginal communities^18^ but are rare (<1%) in vaginal communities in other mammals^53^. Attempts to colonize animal models such as non-human primates^71^ or laboratory mice^29–33^ with human vaginal bacteria have failed to achieve long-term colonization. Key goals of this study were to examine the impact of host environment, microbial seeding, and estrous cycle on the vaginal microbiota in mice with the same genetic background and to establish the influence of vaginal microbiota in pathogen control by using conventional mice and mice exposed to human microbes.

The comparison of the vaginal microbiota across mice of the same genetic background within and between vivaria provides insight into both retained features and variability in mouse models. Samples were collected at different times and sequenced through different pipelines; however, comparable sequencing and intra-colony similarities in Jackson mice at both sites demonstrated that inter-colony differences were not artifacts of this confounding variable. An additional limiting factor is the low sample number of UCSD mice; even so, effect sizes were large enough to determine statistical significance.

Similar to murine fecal communities^45,72^, we observed unique signatures in the otherwise conserved vaginal microbial composition of C57BL/6J mice that gave rise to phylogenetic diversity within and across three distinct facilities. The vaginal microbiota of ^HMb^mice recapitulated the community structure seen in conventional mice characterized by low alpha diversity and dominance by a single taxon in most mice. This finding suggests that host selective pressures drive the vaginal community towards a skewed dominance of a single organism independent of microbial exposure^25–27^. We observed a slight, but significant decrease in ^HMb^mice vaginal pH compared to conventional mice; however, the biological relevance of this difference and how it relates to pathogen colonization warrants further investigation. Clinically, the threshold of pH 4.5 does not affect colonization rates of GBS^73^; yet, GBS enhances virulence via transcriptional regulation in pH 7.5 compared to pH as high as 5.5^74^. Moreover, we discovered high intra-colony variability in cohorts sampled across multiple years. Counter to prior observations^44,75,76^, microbial fluctuation was not explained by seasonal changes or convergence of microbial compositions over the two-year sampling period, but rather was likely due to an accumulation of differential colony maintenance, diet, and environmental stimuli^45,72,77^. Congruent with our study, distinct changes to the vaginal microbiota have also been reported in wild field mice upon captivity^78^. Age has also been associated with changes in vaginal microbiota, with an increase in alpha diversity in post-menopausal women^62,79^. ^HMb^mice exhibited decreased richness in vaginal microbial composition with increased age from weaning to 6 months of age; however, composition beyond breeding range were not evaluated in this study.

Reproductive parameter differences between ^HMb^mice, conventional BCM, and Jackson mice likely resulted from colony management rather than biologic divergences as litter size and number of litters per 6 months were similar. An important limitation of this study is that we did not evaluate microbial composition dynamics in pregnancy, of which little is known in mice. Recent human studies have shown an increase in Shannon diversity towards the end of pregnancy^51^ and similar dynamics were recently described in a longitudinal pregnancy study in conventional mice^80^. The human vaginal microbiota fluctuates over the menstrual cycle following hormone-mediated changes in glycogen availability, a key nutrient for Lactobacilli and other microbes^53,81,82^. Observations in other mammals are mixed; reproductive cycle-associated fluctuations have been reported in some non-human primates, cows, and rats^83–87^ but not other non-human primates, horses, or mini-pigs^88–91^. Unlike conventional mice^26^, we observed correlations between estrous stage and vaginal communities, particularly *Escherichia-*dominant ^h^mCST III-a, in ^HMb^mice. Similar to human studies^58–63^, we observed modest, but significant, fluctuations in alpha (Shannon) and beta diversity (Bray-Curtis) with the lowest alpha diversity occurring during estrus and a high beta-diversity during metestrus. An important caveat is that we qualitatively determined estrous stage based on vaginal cytology and not hormone levels. Additionally, this study may be underpowered to delineate distinct microbial signatures that could be better resolved by daily tracking of a larger number of individual mice.

Despite intra-colony variability, we observed consistent ^h^mCSTs in ^HMb^mice suggesting continuity of dominant microbes over time. Compared to conventional mice, ^HMb^mice were enriched in colonization by *Lactobacillus* spp.; *Lactobacillus* dominance occurred in 2.8% of samples from Jackson mice but 25.3% of samples from ^HMb^mice. However, there remain several limitations to this model. There is likely some “conventionalization” of ^HMb^mice since we detected a *Staphylococcus*-dominant community (^h^mCST V) which is the most common community in conventional C57BL/6J Jackson mice^25,26^. Additionally, ^HMb^mice frequently colonized by *Ligilactobacillus-*dominant communities (^h^mCST II) had OTUs mapping to *L. murinus*. While only a rarely reported human-associated species^49^, *L. murinus* is a homofermentative lactic acid bacterium^92^ isolated from the vaginal tract of wild mice^78^ and gut of conventional C57BL/6J mice^93^, having the genetic capacity for glycogen metabolism^94^ and pathogen inhibition^95^. It is also important to note that, despite identification of ^HMb^mice sequences mapping to *L. crispatus, L. gasseri*, and *L. jensenii*, it is not possible to ascribe the same functionality between the taxa identified in this study and human gut (the source of human microbes for ^HMb^mice) or vaginal species without further characterization. Lastly, another frequently observed community was dominated by *Enterobacteriaceae* including *Escherichia* (^h^mCST III-a). Although *E. coli* are reported in human vaginal samples, they are typically at low relative abundance^96–99^. Importantly, even with overlapping microbial phylogeny, human and ^HMb^mice reproductive tracts remain physiologically dissimilar^100^.

Because the vaginal microbiota is thought to protect against pathogens and microbial-based disorders, we tested the impact of the ^HMb^mice vaginal microbiota of on representative agents of AV and neonatal disease (GBS and UPEC) and BV (*P. bivia*)^66,101^. GBS colonization is correlated with specific taxa including *Staphylococcus* spp., *P. bivia*, and *E. coli* in non-pregnant women^73,98,102^, and a non-*Lactobacillus* dominant microbiota in pregnant women^103^. GBS uterine ascension, a mechanism for pregnancy complications^104–106^, is correlated with *Staphylococcus*-dominant vaginal microbiota in conventional mice^26^. Although there were minimal differences in GBS vaginal burdens between conventional and ^HMb^mice, ^HMb^mice demonstrated lower or undetectable uterine burdens and revealed four vaginal taxa (three with very low abundance) that were inversely correlated with detection of uterine GBS. *L. murinus*, but not *E. coli*, reduced GBS growth in coculture experiments, while exogenous treatment of *E. coli*, but not *L. murinus*, reduced GBS colonization in ^HMb^mice. The discordance between in vitro and in vivo findings could be explained by attenuation of *L. murinus* anti-GBS activity in vivo due to poor colonization, altered competitive ratios, or insufficient production of anti-GBS factors. Alternatively, the in vivo success of exogenous *E. coli* could be explained by *E. coli* outcompeting GBS for key nutrients or attachment to host surfaces or the elicitation of an altered immune response. These studies highlight the complexity of host and microbial factors dictating GBS colonization success and may explain why some probiotics with potent anti-GBS activity in vitro have failed to reduce GBS colonization in clinical trials^107–110^.

^HMb^mice were not consistently resistant to pathobionts compared to conventional mice; ^HMb^mice displayed reduced tissue burdens of *P. bivia* but not UPEC. *P. bivia* uterine burdens in conventional mice were comparable to previous studies^111^ suggesting that ^HMb^mice may actively suppress *P. bivia* uterine ascension or persistence. Interestingly, only *P. bivia*-challenged ^HMb^mice exhibited reduced cytokine levels in comparison to conventional mice. Whether uterine IL-17 was decreased because of reduced *P. bivia* burden or other modulations by the endogenous flora was not determined. No differences in UPEC colonization were seen between ^HMb^mice and conventional mice; tissue burdens were consistent with previous findings^70,112,113^. Counter to other murine gut colonization models^114^, exogenous UPEC did not appear to be negatively impacted by the frequent endogenous vaginal *E. coli* found in ^HMb^mice; however, we did not assess changes to the vaginal microbiome following UPEC inoculation, so it is unknown whether UPEC had any impact on endogenous *Enterobacteriaceae* including *E. coli*. An important limitation of our study is that assessment of host immune responses to these pathobionts was limited to two cytokines. Previous studies demonstrate that GBS and UPEC, but not *P. bivia*, induce vaginal immune responses in conventional mice^68,112,115^, thus further characterization of the ^HMb^mice model, paired with in vitro models such as a human vagina-on-a-chip^116^, is needed to delineate the role of the vaginal microbiota in shaping immune responses to vaginal pathobionts.

Our results reveal the plasticity of the mouse vaginal microbiota in response to environmental exposures, perhaps a more potent driver of variability than host genetics. Additionally, we found microbial drift between cohorts, age, and within individual mice over the course of a week. Even so, questions remain regarding the biologic factors driving rapid changes to the vaginal microbiota in mice. Although not an exact representation of the human vaginal microbiota, the ^HMb^mouse model described here is enriched in *Lactobacillus*-dominant communities and demonstrates the importance of the vaginal microbiota in eliciting immune responses and shaping outcomes of reproductive tract infections. Continued improvement of humanized mouse models will provide a pathway to establish the functional role of the vaginal microbiota in health and disease and serve as an improved preclinical model for microbe-based therapies.

## Methods

### Bacterial strains

GBS COH1 (ATCC BAA-1176), a neonatal meningitis isolate, was grown overnight in Todd-Hewitt Broth (THB) at 37 °C, diluted 1:10 in fresh THB, and grown to mid-log phase (OD_600nm_ = 0.4). Spontaneous streptomycin-resistant mutants of human oral isolate *L. crispatus* (ATCC 33820)^117^ and cystitis isolate UPEC UTI89^118^ were generated by plating an overnight culture on De Man, Rogosa and Sharpe (MRS) agar or Luria Broth (LB) agar, respectively, containing 1000 μg/mL Streptomycin. *L. crispatus* Strep^R^ was grown anaerobically in a Coy anaerobic chamber at 37 °C in MRS with 1000 μg/mL Streptomycin, washed twice with PBS and saved in 5% glycerol working aliquots. UPEC Strep^R^ was grown overnight in LB with 1000 μg/mL Streptomycin and washed twice with PBS prior to use. *Prevotella bivia* ATCC29303 Strep^R^, an endometrial isolate^115^, was grown anaerobically in a Coy anaerobic chamber at 37 °C in Tryptic Soy Broth (TSB) with 5% laked, defibrinated sheep blood for three days. *E. coli* and *L. murinus* were isolated from ^Hmb^mice vaginal swabs plated on MRS agar and grown anaerobically in MRS over one or two days, respectively.

### Animals

Animal experiments were approved by the BCM and University of California San Diego Institutional Animal Care and Use Committees and conducted under accepted veterinary standards and in compliance with all relevant ethical regulations. Mice were given food and water *ad libitum*. Humanized Microbiota mice (^HMb^mice) were maintained as described previously^36^. WT C57BL/6J female mice were purchased from Jackson Labs (#000664) or from C57BL/6J stocks bred at BCM and UCSD. Prior to bacterial infections, mice were acclimated for one week in the biohazard room. Mice were distributed so that each treatment group and timepoint contained a similar age range in mice. Mice ranged in age from 2 to 6 months. At predetermined experimental endpoints day 3 or day 7 post infection, mice were euthanized by C0_2_ according to the *Guide for the Care and Use of Laboratory Animals*^119^ and then cervically dislocated as a secondary measure.

### Vaginal lavage collection and pH assignment

Vaginal lavages were collected using 10 µL of molecular grade water. Water was pipetted into the vaginal tract five times and spotted onto both a strip of Fisher Brand pH paper measuring pH 3.0−5.5 at 0.5 intervals (Lot: 214220) and another strip measuring pH 6−8 at 0.2 intervals (Lot: 211718 A). Values were assigned, unblinded, by three independent researchers. For measurements that sat between two intervals, the median pH was recorded. Final scores were averaged. Only one independent experiment was performed (*n* = 9−10).

### Vaginal swab collection and estrous stage assignment

Vaginal swabs were collected as previously described^65^, resuspended in 100 μL of PBS, and stored at -20 °C. Samples from UCSD conventional mice and Jackson mice were collected and sequenced in the same time frame at UCSD. A second set of Jackson mice were swabbed and sequenced at BCM along with BCM conventional and ^HMb^mice. Wet mounts of vaginal samples were observed under brightfield 100X magnification on an Echo Revolve microscope. Estrous stages were delineated, unblinded, by three independent researchers according to parameters described previously^120,121^ and assigned with a consensus of at least two researchers. Mice were sampled at a single time point (*n* = 2), every three days (*n* = 28), or daily (*n* = 5) over the span of seven days.

### DNA extraction and 16S rRNA V4 amplicon sequencing

DNA from thawed vaginal swabs was extracted using the Quick-DNA Fungal/Bacterial Microprep Kit protocol (Zymo Research) and following manufacturer’s instructions with two deviations: samples were homogenized for 15 min during lysis and DNA was eluted in 20 µL of water. Amplification and sequencing of the V4 region of the 16S rRNA gene were carried out by BCM Center for Metagenomics and Microbiome Research or UCSD Institute for Genomic Medicine using the Illumina 16Sv4 and Illumina MiSeq v2 2x250bp protocols as described^25,26^. Sequences were joined, trimmed to 150 bp reads, and denoised using Deblur through QIIME2 version 2022.8^122^. Operational Taxonomic Units (OTUs) were assigned using Greengenes2 reference tree^48^.

Vaginal sequences of Jackson Labs mice from previous work were downloaded from EBI accession numbers PRJEB25733^26^ and PRJEB49304^25^, and NCBI Sequence Read Archive BioProject accession number PRJNA988548 as supplement to our present study deposited under EBI accession number PRJEB58804 for Fig. 1 and Supplementary Figs. 1,3,5. Since many samples were low biomass, DNA contaminants from sequencing reagents and kits had a substantial impact on the dataset and necessitated filtering of Feature IDs as presented in Supplementary Fig. 2. First, feature IDs that appeared in less than five samples were removed. Second, negative controls from BCM (*n* = 15) and UCSD (*n* = 18) that went through the entire pipeline, from DNA extraction to sequencing, were run through the R package Decontam^123^(R version 4.3.1 (2023-06-16) – “Beagle Scouts”), which identified 27 Feature IDs that were subsequently removed from the feature table. Lastly, the feature table was re-imported into QIIME2 where other abundant contaminants were filtered out.

Alpha diversity (OTUs and Shannon), beta diversity (Bray-Curtis and weighted UniFrac distances analyzed by PERMANOVA and PERMDISP), and differential abundance (ANCOM) tests were carried out in QIIME2^124^. PCoA plots, the only data represented from rarefied tables, were produced by UPGMS clustering using the “diversity beta-rarefaction” command at a rarefaction depth of 100 for 100 iterations. To assign ^h^mCSTs and create heatmaps, hierarchical clustering was performed on the filtered feature table with Ward’s linkage of Euclidean distances using the R package stats^25^. Output files were exported and analyzed in R Studio v1.2.5001 using factoextra^125^, and Phyloseq^126^. Data visualization was performed with GraphPad Prism v9.4.0 (GraphPad Software Inc.).

### Reproductive parameters and data

Jackson Labs data was acquired from the Handbook on Genetically Standardized JAX Mice^57^. BCM C57BL/6 J and ^HMb^mice colony data were sourced from colony managers. Ranges were not provided for all measures. Parameters for Jackson, BCM, and germ-free mice were set as theoretical means against which ^HMb^mice were compared.

### Murine pathogen colonization models and sample analyses

Vaginal colonization studies were conducted as described previously^65,111^. Mice were synchronized with 0.5 mg β-estradiol administered intraperitoneally 24 h prior to inoculation with GBS, UPEC Strep^R^ or *L. crispatus* Strep^R^ (Fig. 5a) and at both 48 h and 24 h prior to inoculation with *P. bivia* Strep^R^. Mice were vaginally inoculated with 10 μL of GBS COH1 (10^7^ CFU), UPEC Strep^R^ (10^7^ CFU), *P. bivia* Strep^R^ (10^6^ CFU), or *L. crispatus* Strep^R^. Vaginal swabs were collected at indicated timepoints and, for pathogen challenge experiments, tissues were harvested on day 3 and/or 7 as previously described^25^. CHROMagar StrepB Select (DRG International Inc.) was used to quantify recovered GBS (identified as pink/mauve colonies). CHROMagar Orientation plates were used to quantify recovered UPEC Strep^R^ (identified as pink colonies). *P. bivia* Strep^R^ was quantified on blood agar containing 1000 mg/mL Streptomycin. *L. crispatus* Strep^R^ was quantified on MRS containing 1000 mg/mL Streptomycin. Only one independent experiment (*n* = 9−10) was performed for *L. crispatus* colonization. For pre-treatment experiments, 0.5 mg β-estradiol was given on day −5 and −2 and either *L. murinus* (10^6^ CFU), *E. coli* (10^6^ CFU), or vehicle were administered on day −4 and −1 before GBS challenge (Fig. 7a). Cardiac puncture was performed to collect serum and to plate for the detection of bacteremia. Blood, swabs and tissues were collected as stated above and plated for GBS CFU on CHROMagar StrepB Select. ELISAs were performed on vaginal swab fluid and tissue homogenates (diluted 1:5 and 1:10 respectively) for mouse MIP-2 and (both diluted at 1:10) IL-17α (R&D Systems) per manufacturers’ instructions.

### In vitro competition assays

GBS, *L. murinus*, and *E. coli* were grown anaerobically in MRS media as cocultures or monocultures at the indicated concentrations. Samples collected at 3 h and 18 h were plated on THB (*L. murinus* competition) or CHROMagar Orientation (*E. coli* competition) and cultured anaerobically at 37 °C for two days.

### Statistics

Data were collected from at least two independent experiments unless otherwise stated. Mean values from independent experiment replicates, or biological replicates, are represented by medians with interquartile ranges or 95% confidence intervals, or box-and-whisker plots with Tukey’s whiskers as indicated in figure legends. Independent vaginal samples, some taken at multiple timepoints from the same mouse, are represented by symbols on PCoA plots. Intra-colony alpha and beta-diversity metrics, and GBS burdens by ^h^mCST were analyzed by Kruskal-Wallis with Dunn’s multiple comparisons test or as paired samples by two-tailed Wilcoxon Rank Sum test. Association between age and OTUs was determined by one-tailed Spearman Correlation. Pathogen burdens and cytokine levels between conventional and ^HMb^mice, pre-treatment conditions, or vaginal and fecal communities, were assessed by two-tailed Mann-Whitney test with corrections for multiple comparisons using the two-stage linear step-up procedure of Benjamini, Krieger and Yekutieli and a false discovery rate (<0.05) where necessary. Inter-colony dissimilarity was analyzed using pairwise PERMANOVA and PERMDISP performed with 999 permutations. Reproductive parameters and competitive indices and were analyzed by a two-tailed one-sample t-test with a theoretical mean of corresponding values from conventional mouse colonies for reproductive data or of 1.0 for in vitro data. ^h^mCST frequencies across estrous stages were compared by two-tailed Chi square test. Coculture and monoculture comparisons were assumed to have parametric distribution and were analyzed by two-way ANOVA with Šídák’s multiple comparisons test. Statistical analyses were performed using GraphPad Prism, v9.4.0. For Mann−Whitney tests with corrections for false discovery and PERMANOVA analyses, adjusted *P* values < 0.1 are shown. Unless otherwise stated, *P* values < 0.05 were considered statistically significant.

## Code availbility

Script and metadata files are accessible at GitHub under project “MouseVaginalMicrobiota-HMb_filtering_CST” at https://github.com/Marlydem/MouseVaginalMicrobiota-HMb_filtering_CST.

### Supplementary information

Supplemental Material

Reporting policy checklist

## Data Availability

Sequencing Data generated by this study is available in EBI under accession number PRJEB58804. Other sequencing data was sourced from EBI accession numbers PRJEB25733 and PRJEB49304, and NCBI Sequence Read Archive under BioProject accession number PRJNA988548.
