# Supplementary material for: Vaginal microbial dynamics and pathogen colonization in a humanized microbiota mouse model
Source: NPJ Biofilms Microbiomes. 2023 Nov 20;9:87. doi: 10.1038/s41522-023-00454-9 (PMC10661851; doi:10.1038/s41522-023-00454-9)
Supplement: Supplementary file 1 — Supplemental Material [file 41522_2023_454_MOESM1_ESM.pdf]

SUPPLEMENTAL MATERIALS

Vaginal microbial dynamics and pathogen colonization in a humanized microbiota mouse model

Marlyd E. Mejia, Vicki Mercado-Evans, Jacob J. Zulk, Samantha Ottinger, Korinna Ruiz, Mallory B. Ballard, Stephanie W. Fowler, Robert A. Britton, and Kathryn A. Patras

Contents:

Supplementary Figures 1-7

Supplementary Tables 1-2

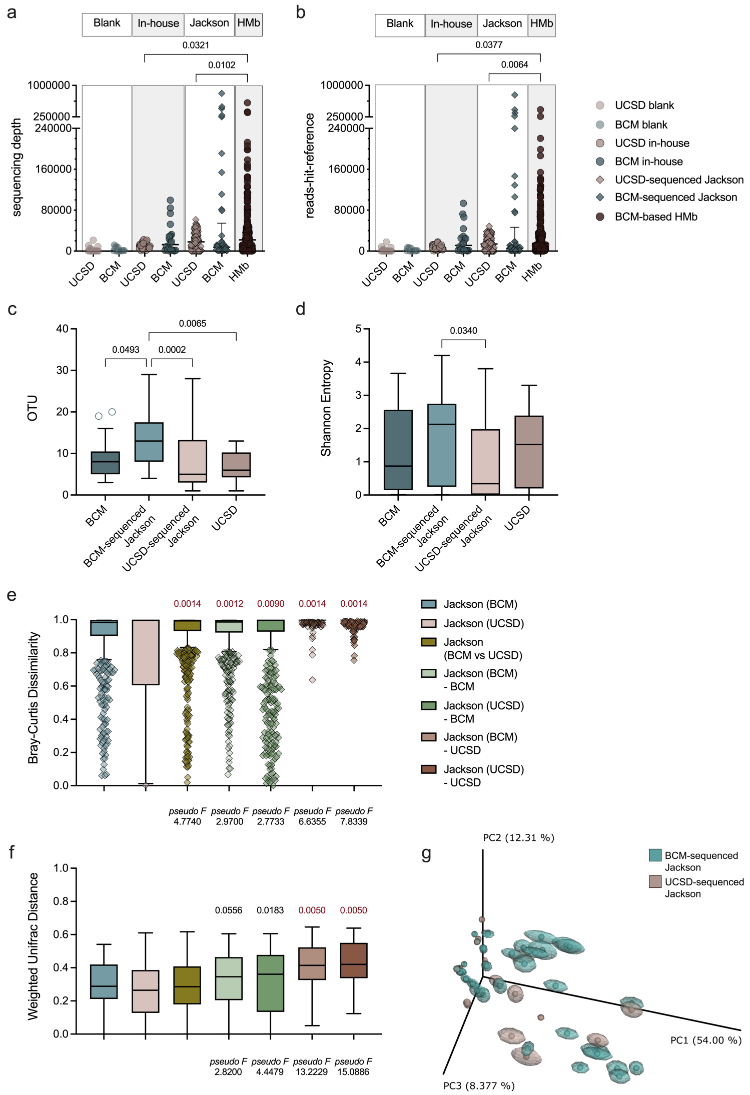

Supplementary Figure 1. Vaginal microbiome data disaggregated by sequencing site. Sequencing output data from vaginal swabs processed in separate sequencing pipelines were compared. **(a)** Read depth and **(b)** reads matched post deblur to the internal Qiime2 reference database for each mouse colony sequenced at BCM or UCSD. Blanks are included per sequencing site (not included in statistical analysis). **(c)** Observed OTUs and **(d)** Shannon entropy of vaginal swab samples. Colony instability and inter-site variation by **(e)** Bray-Curtis or **(f)** weighted normalized UniFrac distances in comparison to either Jackson mice sequenced at BCM or UCSD. **(g)** PCoA of vaginal communities clustered by weighted normalized UniFrac distances between Jackson colonies. Centroids were determined by jackknifed rarefaction (100 reads). Each symbol (a-b, g) represents a unique vaginal swab community/sample with median and interquartile ranges (a-b). Tukey's boxplots are displayed (c-f). Data were analyzed by Kruskal-Wallis with a Dunn's multiple comparison test excluding blanks (a-d) or PERMANOVA followed by PERMDISP (e-f). PERMANOVA *P* values (*P* < 0.1) are colored in red if PERMDISP was also statistically significant (<0.05). All other statistically significant *P* values are reported. Adjusted *P* values < 0.1 are reported for e-f.

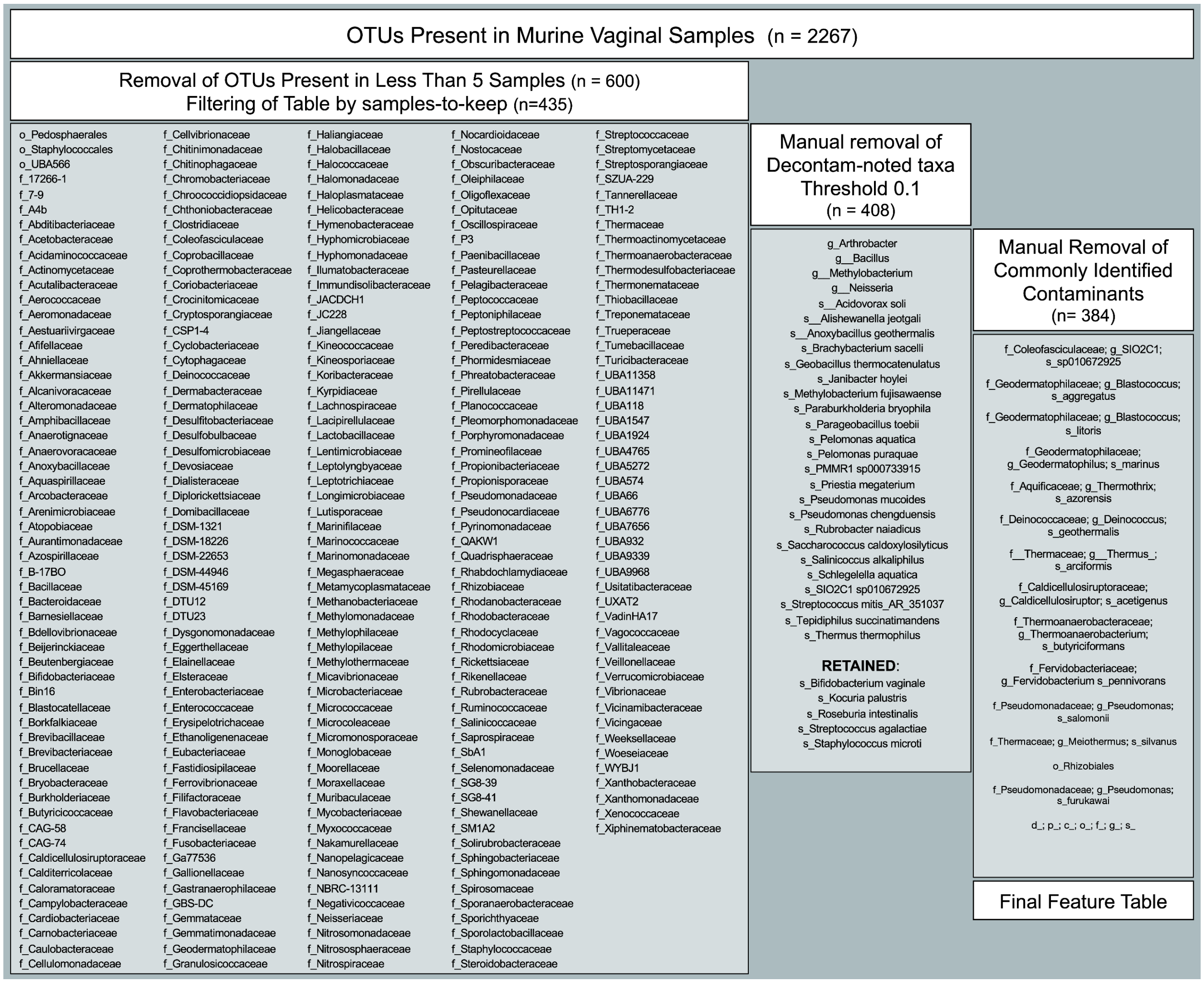

Supplementary Figure 2. Flow chart of contaminant sequence removal to generate feature tables. All studies were merged prior to taxonomic assignment. In the resulting feature table, OTUs that appeared in less than 5 samples were removed in Qiime2 followed by removal of contaminants using Decontam in R. The table was re-imported into Qiime2 for the manual removal of known contaminants.

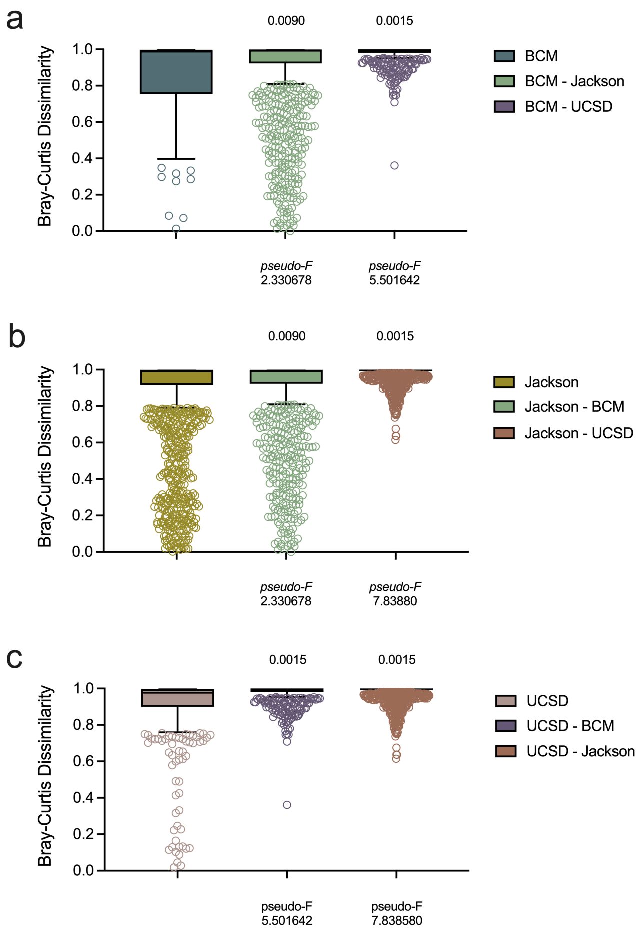

Supplementary Figure 3. The microbial composition of the murine vaginal tract varies between vivaria. Vaginal swabs from mouse colonies raised at BCM (*n* = 21), Jackson Lab (*n* = 30), and UCSD (*n* = 12, mice repeatedly swabbed) were collected and sequenced over the 16S rRNA V4 region. Colony instability and inter-site variation (Bray-Curtis dissimilarity) in comparison to **(a)** BCM, **(b)** Jackson Labs, and **(c)** UCSD. Tukey's boxplots are displayed (a-c). Data were analyzed by PERMANOVA followed by PERMDISP (a-c). PERMANOVA *P* values ($P < 0.1$) are colored in red if PERMDISP was also statistically significant ($P < 0.05$). Adjusted *P* values < 0.1 are reported.

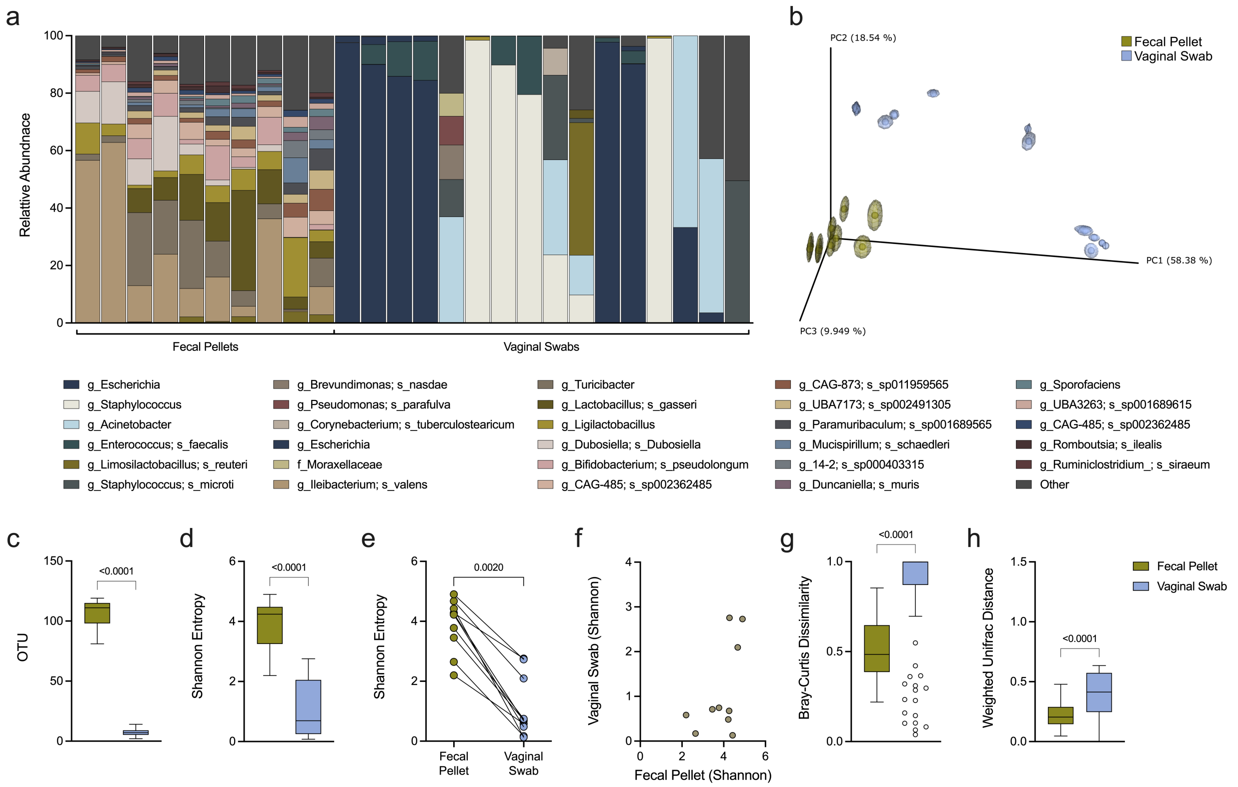

Supplementary Figure 4. ^{HMb}mice have tissue-specific microbial compositions. Vaginal swab and fecal pellet sets were collected from a cohort of ^{HMb}mice (*n* = 16) **(a)** Microbial compositions of fecal pellets (left) and vaginal swabs (right) where the first ten vaginal communities are matched to fecal communities in corresponding order. **(b)** PCoA of vaginal and fecal communities clustered by weighted normalized UniFrac distances. Centroids were determined by jackknifed rarefaction (100 reads). **(c)** Observed OTUs, **(d)** Shannon entropy, and **(e)** pairwise Shannon entropy (*n* = 10) between vaginal and fecal communities. **(f)** Correlation between Shannon entropy in vaginal and fecal samples within mice (*n* = 10). **(g)** Bray-Curtis or **(h)** weighted UniFrac distances from both sample types. Tukey's boxplots (c-d, f-g) or individual samples or comparisons marked as columns (a) or symbols (b, e) are displayed. Data were statistically analyzed by Mann-Whitney (c-d, g-h) or Wilcoxon Rank Sum test (e), or Spearman Correlation (f). Statistically significant *P* values are reported.

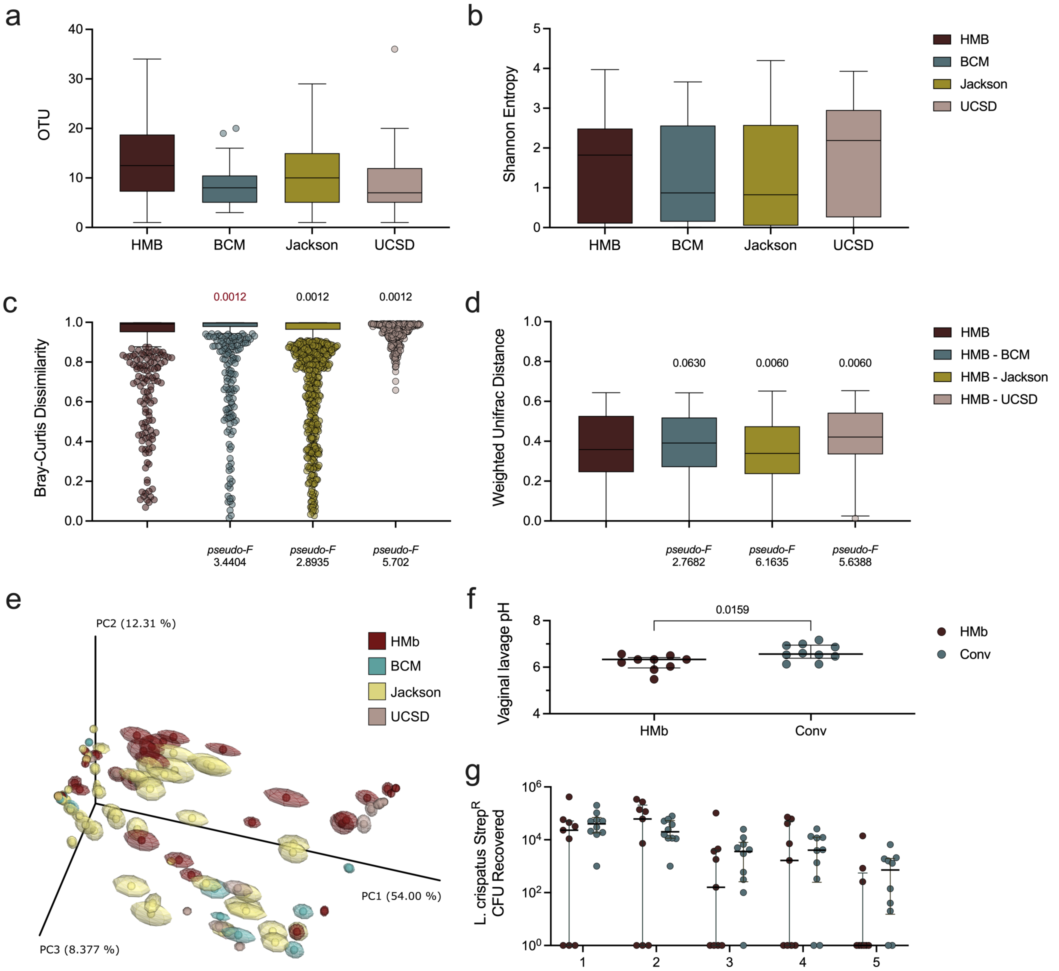

Supplementary Figure 5. ^{HMb}mice display unique and variable vaginal microbiota compared to conventional mice. ^{HMb}mice vaginal compositions were compared to the vaginal microbiota of the conventionally colonized mice from **Fig. 1**. **(a)** Observed OTUs and **(b)** Shannon entropy of vaginal swab samples. HMb colony instability and inter-colony variation by **(c)** Bray-Curtis and **(d)** weighted normalized UniFrac distances in comparison to BCM, Jackson Labs, and UCSD. **(e)** PCoA of vaginal communities clustered by weighted normalized UniFrac distances between colonies. Centroids were determined by jackknifed rarefaction (100 reads). **(f)** Vaginal pH of ^{HMb}mice and conventionally colonized BCM mice determined from vaginal lavage. **(g)** Duration and abundance of Lactobacillus colonization in ^{HMb}mice and conventionally colonized BCM mice. Experiments were performed as one independent replicate (f-g). Tukey's boxplots (a-d) and individual samples marked as symbols (e) or symbols with median and interquartile ranges (f-g) are displayed. Data were statistically analyzed with Kruskal-Wallis with Dunn's multiple comparisons test (a-b), by PERMANOVA followed by PERMDISP (c-d), or Mann-Whitney (f) with corrections for multiple comparisons using the two-stage linear step-up procedure of Benjamini, Krieger and Yekutieli and a false discovery rate (<0.05) (g). PERMANOVA *P* values (P < 0.1) are colored in red if PERMDISP was also statistically significant (P < 0.05). Statistically significant *P* values are reported. Adjusted *P* values < 0.1 are reported for c-d, g.

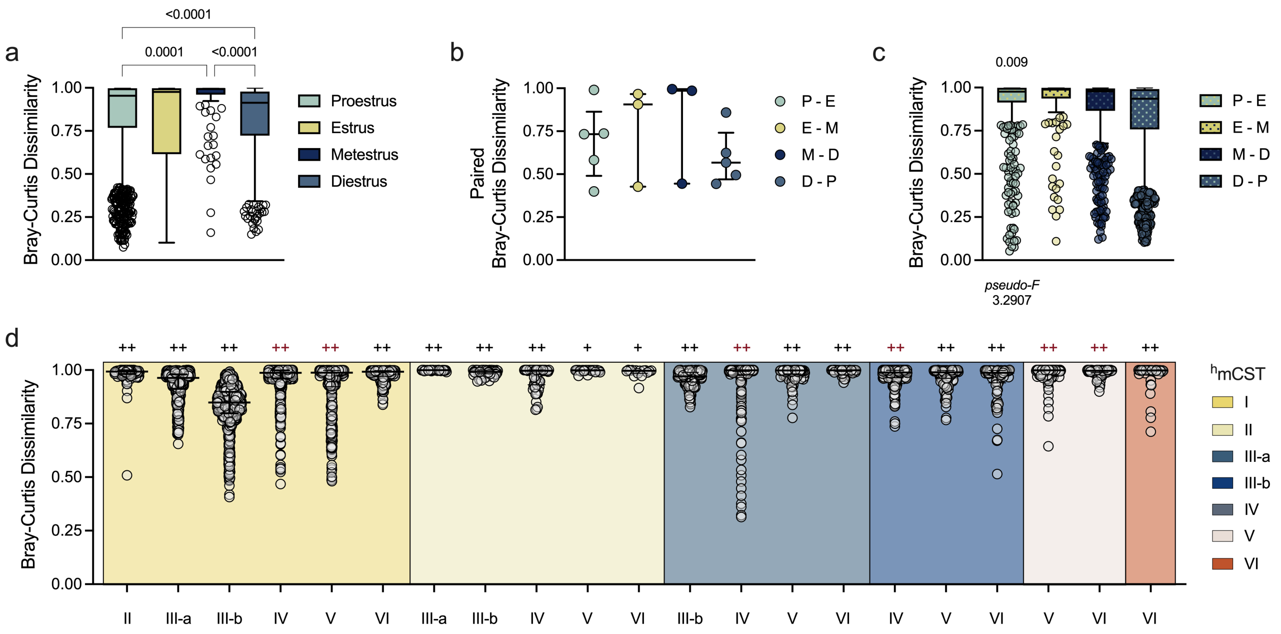

Supplementary Figure 6. Vaginal microbiota dynamics across estrous stages and between ^{h}mCST in ^{HMb}mice. Vaginal swabs were collected at baseline and assigned the host's estrous stage at time of collection. Bray-Curtis dissimilarity of microbial compositions **(a)** among samples categorized in the same estrous stage (n = 35), **(b)** between paired, consecutive samples collected from individual mice, **(c)** between unpaired samples from sequential estrous stages, or **(d)** between ^{h}mCSTs (n = 183 samples). ^{h}mCSTs labeled below are compared to the ^{h}mCST of the corresponding background color. **(d)** Tukey's boxplots (a,c) or individual comparisons marked as symbols with median and interquartile ranges (b,d) are displayed. Data were statistically analyzed by Kruskal-Wallis with Dunn's multiple comparisons test (a-b) or PERMANOVA followed by PERMDISP (c-d). PERMANOVA P values (P < 0.1) are colored in red if PERMDISP was also statistically significant (P < 0.05). All statistically significant P values are reported. $^{+}P < 0.03$; $^{++}P < 0.003$.

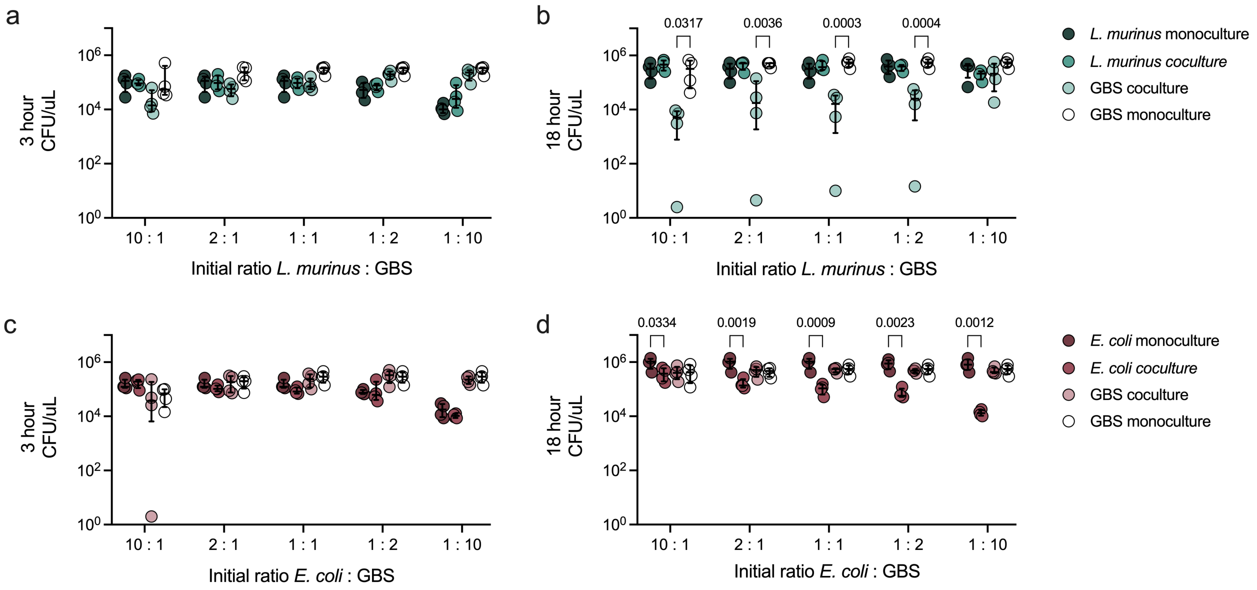

Supplementary Figure 7. Viable CFU recovered from GBS competition experiments with *L. murinus* and *E. coli*. GBS was cocultured with either **(a, b)** *L. murinus* or **(c, d)** *E. coli* at increasing concentrations of GBS. Viable CFU of all microbes in coculture as well as monoculture controls were quantified at **(a, e)** 3 hours and **(b, d)** 18 hours. Individual experimental replicates marked as symbols with median and interquartile ranges are displayed. Comparisons between monoculture and coculture were analyzed by 2-way ANOVA with Šídák's multiple comparison test; statistically significant *P* values are shown.

Supplementary Table 1. *Ligilactobacillus* species candidates for ^{HMb}mice-associated OTUs cross-referenced through BLAST[1]

Taxonomy	OTU[2]	16S v4 Region Sequence	BLAST result
g_ Ligilactobacillus; s_	RS-GCF-910574405.1-NZ-CAJTAQ010000093.1	TACGTAGGTGGCAAGCGTTATCC<br>GGATTTATTGGGCGTAAAGGGAA<br>CGCAGGCGGTCTTTTAAGTCTGAT<br>GTGAAAGCCTTCGGCTTAACCGG<br>AGTAGTGCATTGGAAACTGGGAG<br>ACTTGAGTGCAGAAGAGGAGAGT<br>GGAACTCCATG	Ligilactobacillus animalis<br>Ligilactobacillus murinus<br>Uncultured bacterium<br>(100% identity)
	MJ005-barcode27-umi40855bins-ubs-6	TACGTAGGTGGCAAGCGTTATCC<br>GGATTTATTGGGCGTAAAGGGAA<br>CGCAGGCGGTCTTTTAAGTCTGAT<br>GTGAAAGCCTTCGGCTTAACCGG<br>AGTAGTGCATTGGAAACTGGGAG<br>GCTTGAGTGCAGAAGAGGAGAGT<br>GGAACTCCATG	Uncultured bacterium<br>(100% identity)<br>Uncultured Firmicutes<br>Ligilactobacillus animalis<br>Ligilactobacillus murinus<br>Ligilactobacillus faecis<br>(99.3% identity)
	MJ009-1-barcode35-umi86035bins-ubs-4	TACGTAGGTGGCAAGCGTTATCC<br>GGATTTATTGGGCGTAAAGGGAA<br>CGCAGGCGGTCTTTTAATTCTGAT<br>GTGAAAGCCTTCGGCTTAACCGG<br>AGTAGTGCATTGGAAACTGGGAG<br>ACTTGAGTGCAGAAGAGGAGAGT<br>GGAACTCCATG	Ligilactobacillus animalis<br>Ligilactobacillus murinus<br>Ligilactobacillus faecis<br>Uncultured bacterium<br>(99.3% identity)
	MJ009-2-barcode52-umi43642bins-ubs-14	TACGTAGGTGGCAAGCGTTATCC<br>GGATTTATTGGGCGTAAAGAGAAC<br>GCAGGCGGTCTTTTAAGTCTGATG<br>TGAAAGCCTTCGGCTTAACCGGA<br>GTAGTGCATTGGAAACTGGGAGA<br>CTTGAGTGCAGAAGAGGAGAGTG<br>GAACTCCATG	Ligilactobacillus murinus<br>Uncultured bacterium<br>(100% identity)
	RS-GCF-004793535.1-NZ-SRYK01000136.1	TACGTAGGTGGCAAGCGTTATCC<br>GGATTTATTGGGCGTAAAGGGAA<br>CGCAGGCGGTCTTTTAAGTCTGAT<br>GTGAAAGCCTTCGGCTTACCCGG<br>AGTAGTGCATTGGAAACTGGGAG<br>ACTTGAGTGCAGAAGAGGAGAGT<br>GGAACTCCATG	Uncultured bacterium<br>(100% identity)<br>Ligilactobacillus animalis<br>Ligilactobacillus murinus<br>(99.3% identity)

[1]Timestamp for BLAST search is Sep. 5, 2023 at 11:40:08.

[2]Top two boldened OTUs are the first and second most abundant OTUs shown in **Fig. 4a**. Other *Ligilactobacillus* OTUs in the study, but not displayed Fig. 4a, are noted below the grey bar.

Supplementary Table 2. Statistics[1] on ^{h}mCST compositional comparisons

			Bray-Curtis						Weighted normalized UniFrac					
			PERMANOVA			PERMDISP			PERMANOVA			PERMDISP		
Group 1	Group 2	Sample size	pseudo-F	p-value	q-value	F-value	p-value	q-value	pseudo-F	p-value	q-value	F-value	p-value	q-value
I	II	50	12.433	0.001	0.001	0.908	0.522	0.693	14.459	0.001	0.001	0.017	0.868	0.868
I	III-a	61	38.618	0.001	0.001	0.310	0.567	0.700	441.093	0.001	0.001	5.428	0.019	0.033
I	III-b	64	28.330	0.001	0.001	4.103	0.051	0.153	187.833	0.001	0.001	6.696	0.013	0.027
I	IV	68	22.759	0.001	0.001	106.351	0.001	0.005	28.772	0.001	0.001	147.736	0.001	0.003
I	V	58	25.598	0.001	0.001	13.192	0.001	0.005	85.269	0.001	0.001	3.993	0.050	0.081
I	VI	52	21.418	0.001	0.001	0.026	0.912	0.959	34.302	0.001	0.001	0.155	0.703	0.738
II	III-a	17	6.906	0.001	0.001	0.107	0.641	0.748	331.763	0.002	0.003	3.018	0.003	0.007
II	III-b	20	6.335	0.001	0.001	0.001	0.968	0.968	42.674	0.003	0.004	0.944	0.386	0.450
II	IV	24	1.866	0.002	0.002	19.327	0.099	0.231	2.799	0.017	0.018	18.220	0.003	0.007
II	V	14	4.439	0.004	0.004	0.347	0.528	0.693	42.290	0.005	0.006	2.401	0.074	0.111
II	VI	8	5.299	0.024	0.024	0.216	0.459	0.689	14.915	0.030	0.030	0.358	0.338	0.418
III-a	III-b	31	18.897	0.001	0.001	0.436	0.349	0.590	40.802	0.001	0.001	20.529	0.001	0.003
III-a	IV	35	7.606	0.001	0.001	20.934	0.001	0.005	23.878	0.001	0.001	114.700	0.001	0.003
III-a	V	25	13.372	0.001	0.001	1.666	0.091	0.231	1213.320	0.001	0.001	0.271	0.443	0.490
III-a	VI	19	11.841	0.001	0.001	0.010	0.913	0.959	529.781	0.001	0.001	1.181	0.247	0.341
III-b	IV	38	8.454	0.001	0.001	29.356	0.001	0.005	12.470	0.001	0.001	59.232	0.001	0.003
III-b	V	28	13.076	0.001	0.001	0.944	0.272	0.519	174.170	0.001	0.001	11.502	0.001	0.003
III-b	VI	22	9.870	0.002	0.002	0.671	0.365	0.590	73.378	0.001	0.001	4.886	0.019	0.033
IV	V	32	5.409	0.001	0.001	16.328	0.002	0.008	11.582	0.001	0.001	90.609	0.001	0.003
IV	VI	26	4.015	0.001	0.001	35.181	0.007	0.025	4.942	0.003	0.004	33.970	0.001	0.003
V	VI	16	7.454	0.001	0.001	2.036	0.138	0.290	61.996	0.001	0.001	1.054	0.260	0.341

[1]Pair-wise PERMANOVA and PERMDISP were performed with 999 permutations using Qiime2 package “qiime diversity beta-group-significance”.
