# Supplementary material for: Vaginal microbial dynamics and pathogen colonization in a humanized microbiota mouse model
Source: NPJ Biofilms Microbiomes. 2023 Nov 20;9:87. doi: 10.1038/s41522-023-00454-9 (PMC10661851; doi:10.1038/s41522-023-00454-9)
Supplement: Supplementary file 2 — Reporting policy checklist [file 41522_2023_454_MOESM2_ESM.pdf]

nature portfolio

Corresponding author(s): Kathryn Patras
Last updated by author(s): Nov 2, 2023

Reporting Summary

Nature Portfolio wishes to improve the reproducibility of the work that we publish. This form provides structure for consistency and transparency in reporting. For further information on Nature Portfolio policies, see our Editorial Policies and the Editorial Policy Checklist.

Statistics

For all statistical analyses, confirm that the following items are present in the figure legend, table legend, main text, or Methods section.

n/a	Confirmed	
☐	☒	The exact sample size (n) for each experimental group/condition, given as a discrete number and unit of measurement
☐	☒	A statement on whether measurements were taken from distinct samples or whether the same sample was measured repeatedly
☐	☒	The statistical test(s) used AND whether they are one- or two-sided<br>*Only common tests should be described solely by name; describe more complex techniques in the Methods section.*
☒	☐	A description of all covariates tested
☐	☒	A description of any assumptions or corrections, such as tests of normality and adjustment for multiple comparisons
☒	☐	A full description of the statistical parameters including central tendency (e.g. means) or other basic estimates (e.g. regression coefficient) AND variation (e.g. standard deviation) or associated estimates of uncertainty (e.g. confidence intervals)
☐	☒	For null hypothesis testing, the test statistic (e.g. F, t, r) with confidence intervals, effect sizes, degrees of freedom and P value noted<br>*Give P values as exact values whenever suitable.*
☒	☐	For Bayesian analysis, information on the choice of priors and Markov chain Monte Carlo settings
☐	☒	For hierarchical and complex designs, identification of the appropriate level for tests and full reporting of outcomes
☐	☒	Estimates of effect sizes (e.g. Cohen's d, Pearson's r), indicating how they were calculated

Our web collection on statistics for biologists contains articles on many of the points above.

Software and code

Policy information about availability of computer code

Data collection	Sequencing data was collected using the Illumina MiSeq platform.
Data analysis	Software for data analyses included QIIME2 version 2022.8, Greengenes2 reference tree, R version 4.3.1 (2023-06-16) – "Beagle Scouts", R Studio v1.2.5001 and R packages Decontam, factoextra, Phyloseq and R package stats. Data visualization was performed with GraphPad Prism v9.4.0.

For manuscripts utilizing custom algorithms or software that are central to the research but not yet described in published literature, software must be made available to editors and reviewers. We strongly encourage code deposition in a community repository (e.g. GitHub). See the Nature Portfolio guidelines for submitting code & software for further information.

Data

Policy information about availability of data

All manuscripts must include a data availability statement. This statement should provide the following information, where applicable:

- Accession codes, unique identifiers, or web links for publicly available datasets
- A description of any restrictions on data availability
- For clinical datasets or third party data, please ensure that the statement adheres to our policy

Sequencing Data generated by this study is available in EBI under accession number PRJEB58804. Other sequencing data was sourced from EBI accession numbers

PRJEB25733 and PRJEB49304, and NCBI Sequence Read Archive under BioProject accession number PRJNA988548. Scripts are accessible at GitHub under project "MouseVaginalMicrobiota-HMb_filtering_CST".

Research involving human participants, their data, or biological material

Policy information about studies with human participants or human data. See also policy information about sex, gender (identity/presentation), and sexual orientation and race, ethnicity and racism.

Reporting on sex and gender	Not applicable.
Reporting on race, ethnicity, or other socially relevant groupings	Not applicable.
Population characteristics	Not applicable.
Recruitment	Not applicable.
Ethics oversight	Not applicable.

Note that full information on the approval of the study protocol must also be provided in the manuscript.

Field-specific reporting

Please select the one below that is the best fit for your research. If you are not sure, read the appropriate sections before making your selection.

☒ Life sciences ☐ Behavioural & social sciences ☐ Ecological, evolutionary & environmental sciences

For a reference copy of the document with all sections, see nature.com/documents/nr-reporting-summary-flat.pdf

Life sciences study design

All studies must disclose on these points even when the disclosure is negative.

Sample size	No statistical methods were used to predetermine sample size; however, sample sizes were similar to our prior work which had sufficient power to determine statistical differences for multiple parameters. Number of animals in part was determined by availability of mice from the breeding colony. Only females were used for experiments due to the focus on the vaginal microbiota.
Data exclusions	No data were excluded from primary datasets of cohorts/experiments used in this manuscript. However, for thorough comparison of sequencing pipelines from different institutions in Figure S1, sequences from other cohorts of mice sampled at UCSD under the same conditions were included to increase power in detecting differences. Such sequencing data are referenced with appropriate accession numbers in the data availability statement but are not carried through beyond Figure S1.
Replication	In vitro experiments were replicated independently four times with results combined prior to analyses. In vivo experiments were replicated at least two times independent with results combined prior to analyses. Only Lactobacillus crispatus colonization and pH testing were performed once as described in the methods and figure legend.
Randomization	All animals were randomly assigned to treatment groups.
Blinding	Blinding was not possible in this study for estrous staging and pH determination as mouse identity was evident to handlers. However, bias was minimized by inclusion of three independent investigators for determination, and determination prior to any other results from the experiments (sequencing) being analyzed.

Reporting for specific materials, systems and methods

We require information from authors about some types of materials, experimental systems and methods used in many studies. Here, indicate whether each material, system or method listed is relevant to your study. If you are not sure if a list item applies to your research, read the appropriate section before selecting a response.

Materials & experimental systems

n/a	Involved in the study
☒	☐ Antibodies
☒	☐ Eukaryotic cell lines
☒	☐ Palaeontology and archaeology
☐	☒ Animals and other organisms
☒	☐ Clinical data
☒	☐ Dual use research of concern
☒	☐ Plants

Methods

n/a	Involved in the study
☒	☐ ChIP-seq
☒	☐ Flow cytometry
☒	☐ MRI-based neuroimaging

Animals and other research organisms

Policy information about studies involving animals; ARRIVE guidelines recommended for reporting animal research, and Sex and Gender in Research

Laboratory animals	WT C57BL/6J female mice were purchased from Jackson Labs (#000664) or from C57BL/6J stocks bred at BCM and UCSD. Hmb mice (germ-free C57BL/6J mice colonized with human fecal microbes) were bred and maintained at BCM. Mice ranged in age from 2-6 months.
Wild animals	The study did not use wild animals.
Reporting on sex	Only female mice were used for these studies due to the biological irrelevance of males for vaginal microbiome studies.
Field-collected samples	The study did not involve field-collected samples.
Ethics oversight	Animal experiments were approved by the Baylor College of Medicine and University of California San Diego Institutional Animal Care and Use Committees and conducted under accepted veterinary standards and in compliance with all relevant ethical regulations.

Note that full information on the approval of the study protocol must also be provided in the manuscript.
